# Global patterns are key to understanding macroevolution

**DOI:** 10.1038/s42003-026-11118-7

**Published:** 2026-10-08

**Authors:** Zhen Guo, Wolfgang Kiessling

**Affiliations:** GeoZentrum Nordbayern, Department of Geography and Geosciences, Friedrich-Alexander-Universität Erlangen-Nürnberg, Loewenichstraße 28, Erlangen, Germany

**Keywords:** Palaeontology, Biodiversity

## Abstract

Regional diversification patterns are increasingly being used to investigate spatial heterogeneity in biodiversity evolution and its drivers. However, estimating and interpreting regional diversification rates is complicated by the fact that regional diversity fluctuations reflect multiple biological processes and may be more sensitive to temporal sampling bias than global patterns. Here, we assess the biological fidelity and sampling sensitivity of regional diversification rates using fossil occurrences of marine invertebrates. We show that immigrations and extirpations account for up to 76% of apparent plate-level generic turnover. Moreover, regional diversity fluctuations are more strongly shaped by preservation, sampling, and tectono-sedimentary history than global patterns. In widely distributed groups, global analyses integrate data across regions and environments, dampen region-specific biases, and recover a higher proportion of shared biological signal. Global analyses therefore remain indispensable for inferring macroevolutionary dynamics over deep time.

## Introduction

An accurate reconstruction of deep-time diversification patterns is a prerequisite for identifying their underlying drivers. Over the past half century, analyses of the global fossil record have enabled the identification of important macroevolutionary patterns, such as the major diversification episodes, the three Great Evolutionary Faunas, and the ‘Big Five’ Phanerozoic mass extinctions^1–6^. These discoveries formed the foundation for subsequent research into the mechanisms underlying macroevolutionary dynamics^7–11^. Nevertheless, diversity and the processes driving diversification are, and have always been, spatially structured^12–17^. Global patterns, which represent aggregated signals across regions, may therefore obscure processes operating at regional scales. Moreover, a growing number of studies have shown that fossil sampling is spatially heterogeneous, further complicating the interpretation of global patterns^13,14,16,17^. These concerns have stimulated growing interest in the spatial structure of macroevolutionary patterns and placed increasing emphasis on analyses conducted at regional scales^16–20^. In some studies, the validity of global patterns has even been questioned^16,17^.

However, estimating and interpreting diversification rates at the regional scale is inherently more complex and potentially more sensitive to bias than at global scale. First, while global patterns necessarily reflect global diversification dynamics (i.e., origination and extinction), regional first and last appearances reflect both diversification dynamics and regional range dynamics (i.e., immigration and extirpation). This distinction is important because environmental and ecological drivers of range shifts are not usually the same as those of origination and global extinction^21^, but most available methods do not explicitly separate these processes^22,23^. Second, both diversification and range dynamics are further filtered by preservation and sampling. Numerous studies have demonstrated strong correlations between fossil diversity and rock availability^24–34^, and the initiation or truncation of sedimentary successions may generate flawed originations and extinctions in the fossil record^35–40^. Such effects may be amplified in regional datasets, which typically encompass fewer depositional settings and are more strongly influenced by idiosyncratic tectonic, sedimentary, and sampling histories. Indeed, the explanatory power of the rock record on biodiversity is greater within some regions than at larger scales^27,34^. Although these issues are well recognised, the extent to which regional diversification rates are generally affected by range dynamics, preservation and sampling biases has not previously been quantified.

Here, we assess the biological significance of regional diversification rates derived from fossil occurrence data. We focus on three major groups of marine invertebrates (Bivalvia, Brachiopoda, Cephalopoda), which have relatively complete global fossil records^41,42^. In this study, regional diversification rates refer to rates calculated from first and last appearances of genera at regional scale, as in some previous studies^16,17,43,44^. Such rates may conflate true diversification dynamics with range dynamics. We used tectonic plates to delineate geographic regions (see Material and Methods). In these regions, we quantified the proportion of first and last appearances that may reflect range expansion or shrinkage as opposed to in situ origination or global extinction. We then evaluated whether apparent regional diversity fluctuations coincided with changes in the availability of fossil-bearing localities. Using marine bivalves as an example, we further illustrated how apparent regional diversification rates can be shaped by these processes, and how the global dataset can mitigate some of these region-specific biases. Our aim is not to diminish the value of regional studies, but to clarify the biases that should be considered when estimating and interpreting regional diversification patterns.

## Results

### A large proportion of regional generic turnover reflects range dynamics

**Fig. 1:**
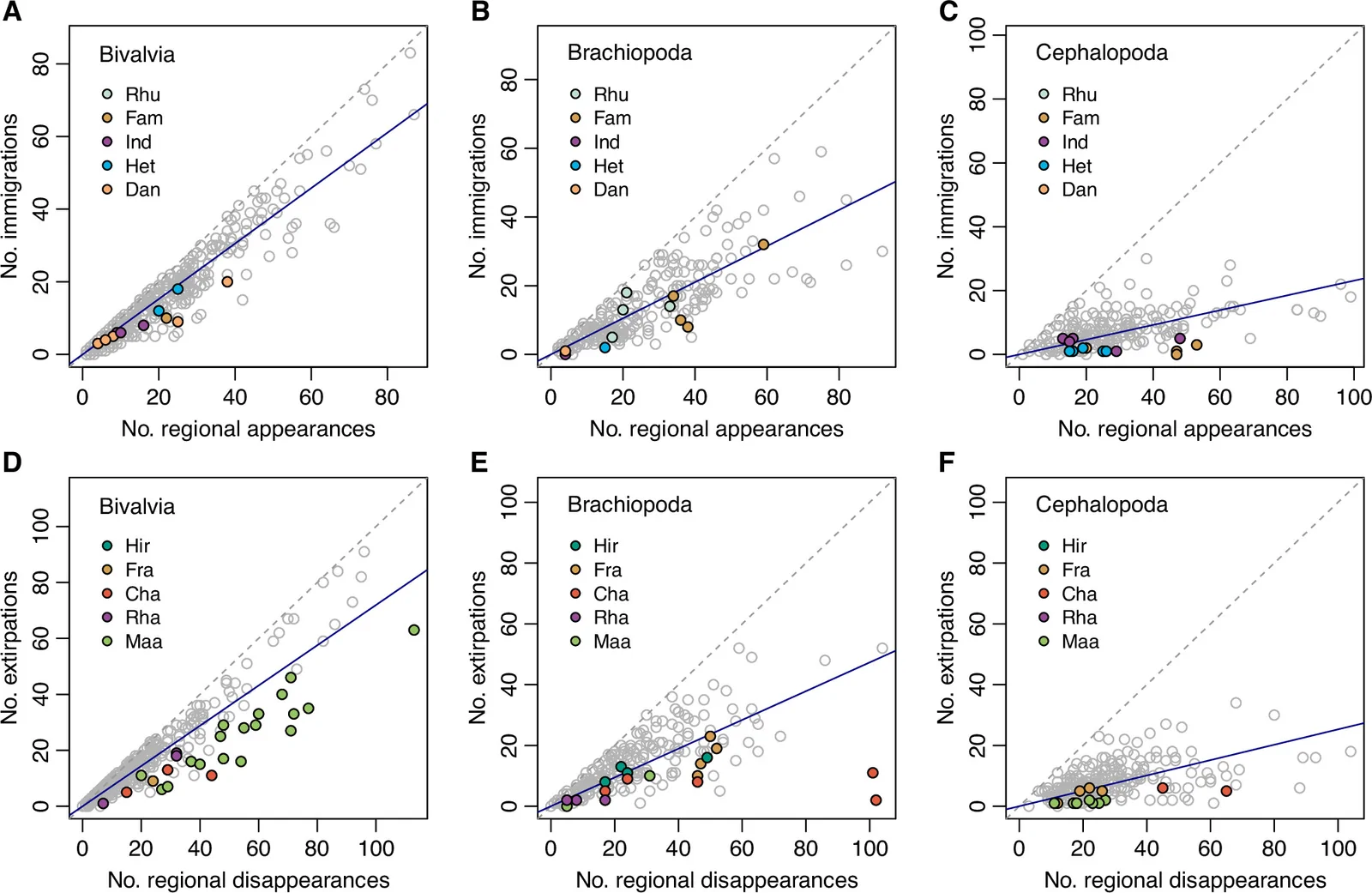
Many regional appearances and disappearances reflect range shifts rather than origination and extinction. **A–C** numbers of regional first appearances and immigrations for bivalves, brachiopods, and cephalopods, respectively. **D–F** numbers of regional last appearances and extirpations for the three groups. Each dot represents data from a specific tectonic plate in a specific stage. For example, 44 bivalve genera last appeared in South China during the Changhsingian, of which 11 were recorded on other plates in younger stages; the coordinate of this data point is therefore (44, 11) in **D**. Least-squares fits were applied to illustrate the proportion of such events (blue lines; intercept constrained to zero). However, their proportions are underestimated (see text). Data from post-mass-extinction intervals in **A**–**C** and from mass extinction intervals in **D**–**F** are highlighted with coloured circles; grey circles denote all other intervals. Grey dashed lines have a slope of one. Rhu, Rhuddanian; Fam, Famennian; Ind, Induan; Het, Hettangian; Dan, Danian; Hir, Hirnantian; Fra, Frasnian; Cha, Changhsingian; Rha, Rhaetian; Maa, Maastrichtian.

To investigate how many apparent regional generic turnovers represent ‘true’ originations (i.e., in situ originations) and extinctions (i.e., global extinctions), or range shifts (i.e., immigrations and extirpations), we compared the first and last appearances of taxa at the regional and global scales. The first and last appearances of genera on a given plate are defined as ‘regional events’ regardless of plate dimensions. Similar events have been treated as ‘regional originations and extinctions’ in some previous studies and used to estimate regional diversification rates^16,17,43,44^. Comparison between the regional and global datasets shows that many regional appearances occurred at least one stage after the global first appearance of the same genus, whereas many regional disappearances occurred at least one stage before its global last appearance (Fig. 1; Table S1). These regional events are here referred to as immigrations and extirpations, although they may also reflect artefacts of preservation and sampling.

A substantial proportion of regional first and last appearances represent immigrations and extirpations, respectively, ranging from 23% to 76% across clades, indicating that range dynamics are important components of apparent regional turnover (Fig. 1). Because range shifts were identified by comparison with the global dataset, their proportions are partly influenced by the degree of faunal endemism: plates containing higher proportions of endemic taxa tend to exhibit lower proportions of immigrations and extirpations (Fig. S5). These proportions also showed weak and often negative correlations with plate size (Spearman’s ρ = −0.21 to 0.01 across clades; Fig. S6), suggesting that larger regions are more likely to capture the global origination and extinction times of taxa. In addition, the proportion of extirpations is lower during intervals of high global extinction intensity (Spearman’s ρ = −0.48 to −0.25), indicating that regional disappearances are more likely to coincide with global extinctions during major extinction episodes (Fig. 1, S4; Table S1). Similarly, the proportion of immigrations tends to be lower following mass extinctions (Spearman’s ρ = −0.37 to −0.35; Fig. 1, S4; Table S1).

The estimated contribution of immigrations and extirpations to regional diversity turnover varies markedly among clades, whether measured as weighted ratios across all plate-stage combinations or as slopes of the fitted relationships in Fig. 1. Bivalves show the highest values, with both metrics ranging from 0.72 to 0.76. Cephalopods show the lowest values, ranging from 0.23 to 0.28, whereas brachiopods are intermediate, ranging from 0.47 to 0.55 (Table S1). To explore the factors underlying these differences, we calculated genus durations, sampling rates, and geographic ranges for the three focal groups (see Methods for details of these metrics). There are pronounced differences in duration and sampling rate among groups (Fig. S11). Bivalves have the longest genus durations (median: 16.67 Myr) and the lowest sampling rates (0.78 occurrences Myr^-1^), whereas cephalopods have the shortest durations (5.82 Myr) and the highest sampling rates (1.14 occurrences Myr^-1^); brachiopods show intermediate values (11.52 Myr and 0.85 occurrences Myr^-1^, respectively). Geographic ranges are similar among the three groups, although bivalves tend to have slightly larger ranges (3,623, 3,008, and 2,981 km for bivalves, brachiopods, and cephalopods, respectively). The long durations, low sampling rates and wide geographical ranges of bivalves imply that a bivalve genus is more likely to disappear from one plate while persisting elsewhere, or to have originated earlier outside the focal plate, thereby elevating the proportions of immigration and extirpations. The high evolutionary rates^45^, readily identifiable morphologies and high dispersal abilities of cephalopods have contributed to their widespread use as index fossils. Consequently, the regional first and last appearances of cephalopods matche best their global stratigraphical ranges.

The proportions of immigrations and extirpations reported here should be regarded as conservative estimates. New taxa originate in geographically restricted areas and may subsequently expand into other regions. As the temporal resolution of PBDB data is generally limited to stage levels, the exact place and time of origin cannot reliably be traced and the first appearance of a genus may be recorded on multiple plates within the same stage. Such cases were not counted as immigrations, even though some of them must represent later regional arrivals within that stage. This underestimation may be particularly pronounced for cephalopods, which have short durations but broad geographic distributions, suggesting rapid dispersal after origination. The same issue also contributes to the lower proportion of immigrations in the aftermath of mass extinctions, when newly originated taxa rapidly migrated across regions^46,47^. It is possible that regional extinctions occurred synchronously, but some extirpations cannot be detected because of limited temporal resolution.

The high proportions of immigration and extirpation contributing to regional generic turnover are noteworthy. Origination and extinction rates are not biologically equivalent to immigration and extirpation rates, and the environmental or ecological drivers of these processes may differ (e.g., refs. ^21,48–50^; see below). Therefore, driver-seeking analyses based on apparent regional turnover (i.e., raw regional appearances and disappearances) require caution. Failure to distinguish diversification from range dynamics may lead to misleading inferences about the processes shaping regional turnover.

### Sampling and sedimentary history influence apparent turnover signals

We assessed the influence of sampling on regional diversity patterns by quantifying how many regional events could be associated with changes in the availability of fossil-bearing localities. Regional events were classified as ‘sampling-associated’ when generic appearances or disappearances coincided with the emergence or loss of sampled fossil localities in equal-area grid cells (Fig. S3). Importantly, this classification does not imply that these events were caused solely by preservation or sampling bias. Changes in the availability of fossil-bearing localities may reflect tectono-sedimentary evolution and facies shifts that simultaneously alter both the distribution of suitable habitats and the probability of fossil preservation. For example, when a depositional environment becomes unsuitable for a taxon, the taxon may be locally extirpated or track its preferred habitat into another area that is not sampled, while the original fossil-bearing facies also disappears. Ecological habitat loss and loss of the sampling window can therefore generate the same observed pattern and may represent a common-cause effect^39^. Because these mechanisms cannot be distinguished reliably with our data, we grouped such events operationally as ‘sampling-associated’, while recognising that some may reflect genuine ecologically driven range shifts or extirpations.

**Fig. 2:**
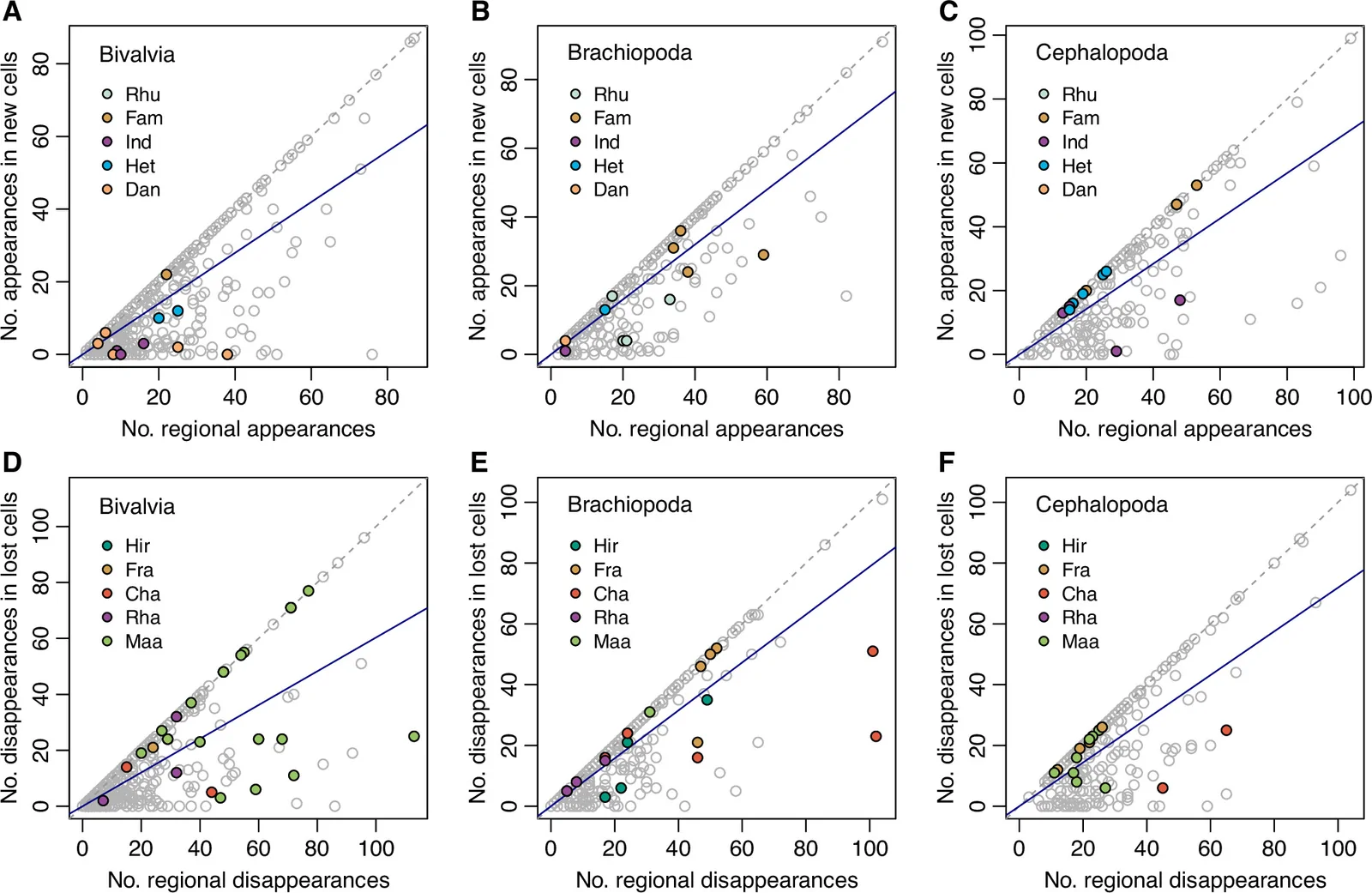
Regional appearances and disappearances are frequently associated with sampling bias. **A–C** total regional first appearances versus first appearances in newly sampled cells. **D–F** total regional last appearances versus last appearances in subsequently unsampled cells. Each dot represents a certain plate of a certain stage. For example, in **D**, 44 bivalve genera last appeared in South China in the Changhsingian stage, and the cells of five genus occurrences were not sampled in the next stage (Induan); the coordinate of this data point is therefore (44, 5). Least-squares fits were applied to illustrate the proportion of such events (blue lines; intercept constrained to zero). Data from post-mass-extinction intervals in **A**–**C** and from mass extinction intervals in **D**–**F** are highlighted with coloured circles; grey circles denote all other intervals. Grey dashed lines have a slope of one. The edge length of hexagonal equal area grid cells is ~123 km. Abbreviations as in Fig. 1.

Our results show that a substantial fraction of regional events is indeed sampling-associated (Fig. 2). Notably, the proportions of sampling-associated events are relatively high across the three groups (range of weighted ratios and slopes of fitted lines: 0.60–0.80; Table S2), indicating that even the cephalopod fossil record, despite its relatively high sampling intensity, is strongly influenced by local environmental shifts and sampling bias. The proportions of these events also show a weak negative correlation with plate size (Spearman’s ρ = −0.30 to −0.03; Fig. S7), suggesting that larger regions tend to buffer taxon losses or gains associated with shifts in fossil localities. The global dataset is also affected by sampling bias, as shown by earlier studies^24–26,38,39^, but for clades analysed here, the proportion of sampling-associated originations and extinctions is slightly lower (0.51–0.74; Table S3), and extreme proportions approaching one are less frequent (Fig. S8). Sensitivity analyses using larger grid cells result in lower numbers and proportions of sampling-related events, but the overall patterns remain unchanged (Fig. S9, S10; Table S4, S5).

**Fig. 3:**
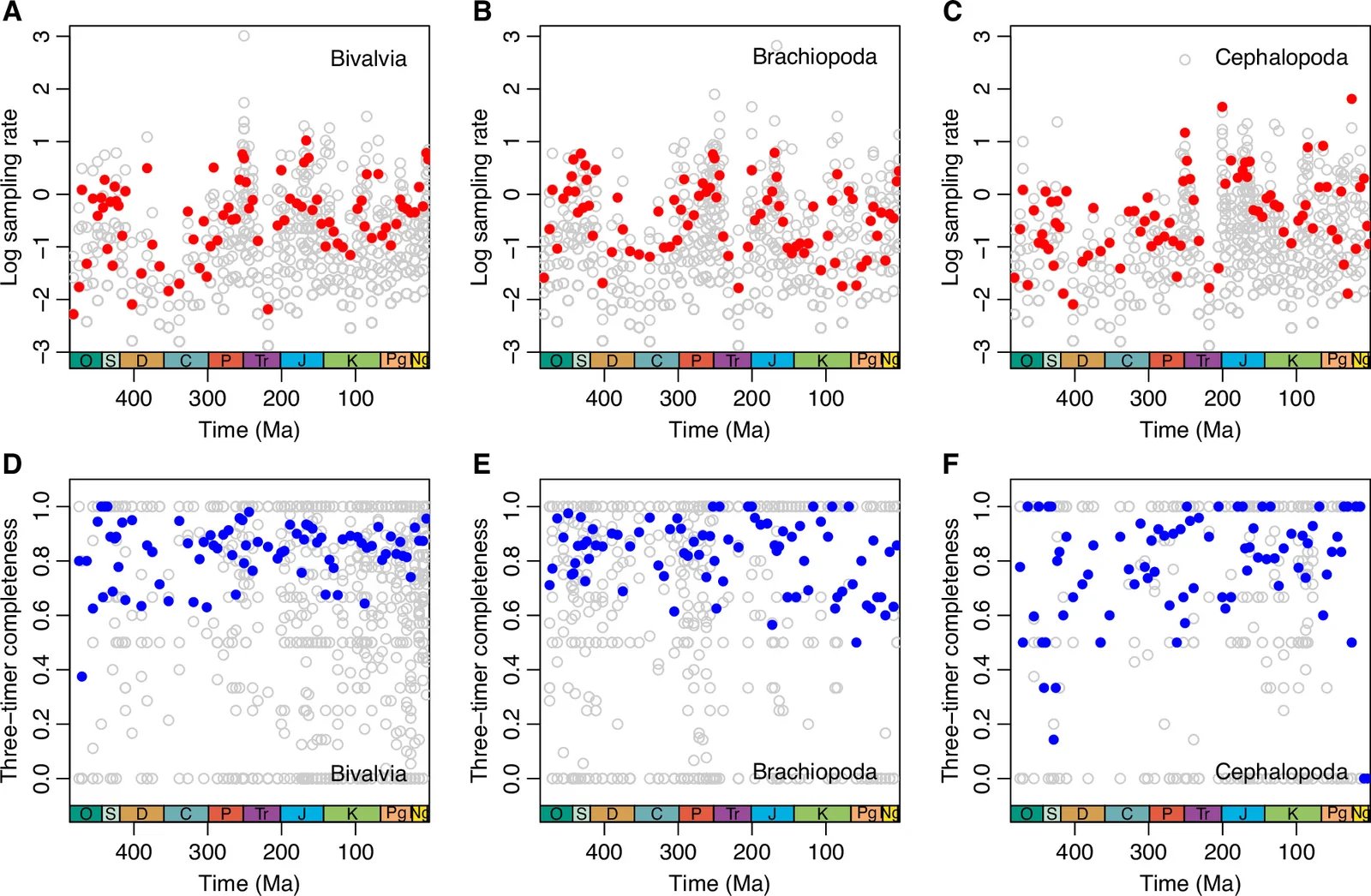
Global sampling is generally more complete than regional sampling. **A**–**C** sampling rate (number of occurrences per million years) for bivalves, brachiopods, and cephalopods. Sampling rates are log-transformed; regional sampling rates of zero (i.e., intervals with no fossil occurrences) are omitted. **D**–**F** three-timer sampling completeness. In all panels, open circles represent regional values and solid dots represent values of the global dataset. Although some regions show higher sampling rates or completeness than the global dataset in particular stages, the global dataset generally exhibits better sampling than regional datasets overall. This difference is supported by one-sided Mann-Whitney tests: *p* < 0.003 in **A**–**E** and *p* = 0.053 in **F**. Sample sizes for the global dataset are *n* = 81, 81, 81, 79, 79, and 79 in **A**–**F**, respectively; sample sizes for regional datasets are *n* = 1694, 1309, 1420, 743, 489, and 353 in **A**–**F**, respectively. See Table S6 for numerical comparisons.

Sampling rates and sampling completeness in the global occurrence datasets are also greater than in regional datasets. Although global datasets contain more occurrences overall, their sampling rates and completeness are not by design greater than those of regional subsets. When a clade exhibits some degree of biogeographical differentiation, a well-sampled region can have high sampling rates and completeness, whereas global estimates may be reduced by poorly sampled regions. Sampling metrics varied greatly through time (Fig. 3). Median regional sampling rates of our three focal groups (0.26–0.27) are much lower than those of the global dataset (0.65–0.71) (Table S6). Global datasets also exhibit higher median sampling completeness (0.81–0.86) than that on individual plates (0.66–0.71) (Table S6). Although some plates have higher sampling rates or completeness than the global datasets in certain stages, overall, the global datasets are expectedly more complete and more continuous than regional datasets (one-sided Mann-Whitney test: *p* < 0.003, except for cephalopod sampling completeness, *p* = 0.053; Fig. 3). The advantage of global integration suggests that the analysed clades have relatively broad geographic distributions and many taxa are shared among regions.

### Bivalves showcase the discrepancy between regional and global diversification rates

**Fig. 4:**
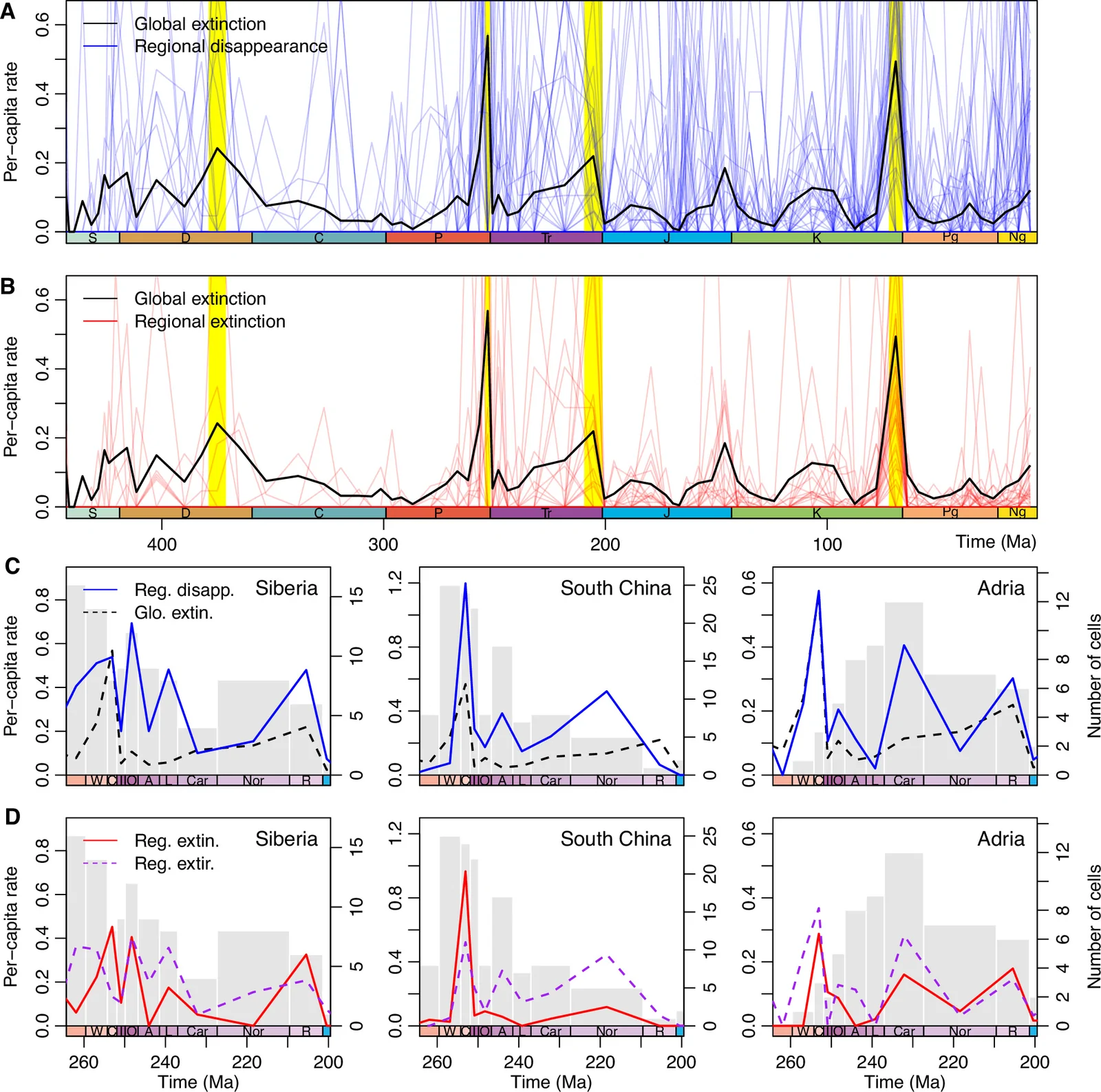
Regional extinction patterns can differ substantially from the global composite because of range dynamics and sampling variation. **A** global extinction rates and regional disappearance rates in marine bivalves from the Silurian to the Miocene. Blue lines represent disappearance rates for individual plates, based on regional last appearances without distinguishing final extinction from extirpation; black line denotes the global extinction rate. **B** comparison of global and regional extinction rates. Red lines represent regional extinction rates; extirpations were not included in the calculation. Mass-extinction stages are highlighted by yellow bars in **A**, **B**. **C** regional disappearance rates (blue solid lines) and global extinction rates (black dashed lines) from the Capitanian to Hettangian for the Siberian craton, South China, and Adriatic plate, respectively. **D** regional extinction rates (red solid lines) and extirpation rates (purple dashed lines) for the three plates. Grey bars in C and D indicate the number of fossil-bearing cells on each plate. Abbreviations in A and B: O, Ordovician; S, Silurian; D, Devonian; C, Carboniferous; P, Permian; Tr, Triassic; J, Jurassic; K, Cretaceous; Pg, Paleogene; Ng, Neogene. Abbreviations in C and D: W, Wuchiapingian; C, Changhsingian; I, Induan; O, Olenekian; A, Anisian; L, Ladinian; Car, Carnian; Nor, Norian; R, Rhaetian; Reg., regional; Glo., global; disapp., disappearance; extin, extinction; extir, extirpation.

Discrepancies between plate-level and global diversification rates in marine bivalves illustrate how inferred regional diversification dynamics can be influenced by range dynamics, regional sedimentary change, and local sampling bias (Fig. 4). The global extinction rate of bivalves is similar to rates reported for other marine groups^3,4,51,52^, with pronounced peaks during the mass extinctions (Fig. 4A). However, regional disappearance rates vary wildly and often exhibit high values during intervals when the global extinction rate was low (Fig. 4A). After separating extirpations from final extinctions, regional extinction rates show less intense fluctuations during background times, further confirming that many regional disappearances represent extirpations. Nevertheless, several erratic peaks remained evident (Fig. 4B), indicating the spatial heterogeneity of observed extinction rates.

Three relatively well-sampled regions exhibit very different patterns of apparent disappearance rates during the Triassic (Fig. 4C). On the Siberian craton, regional extinction rates show clear peaks associated with both the end-Permian and end-Triassic mass extinctions, but also exhibit elevated values during the Wuchiapingian, Olenekian, and Ladinian stages (Fig. 4C). In South China, regional disappearance rates record a strong signal of the end-Permian but little evidence for the end-Triassic mass extinction, with elevated rates in the Anisian and Norian stages (Fig. 4C). The rates in the Adriatic (Apulian) plate capture both the end-Permian and end-Triassic mass extinctions, but show a prominent Carnian peak that is not prominent in the global pattern (Fig. 4C).

Decomposing regional disappearances into extirpations and extinctions, together with changes in the number of sampled fossil localities, helps explain these discrepancies (Fig. 4D). Both the end-Permian and end-Triassic mass extinctions were driven by severe environmental perturbations and had global-scale impacts^53–55^. However, the decline in sampled fossil localities in the Changhsingian of Siberia and the Late Triassic marine regression in South China shifted a substantial proportion of regional disappearances to stages preceding the corresponding global mass extinctions (Fig. 4D). Consequently, many extirpations predated the mass-extinction intervals. The Carnian Pluvial Episode also represented a global environmental perturbation that is thought to have profoundly affected animal evolution^56^. Carnian extinction rates exhibit pronounced spatial heterogeneity^17^ (Fig. 4C, D), and this pattern partly reflects contrasting sampling regimes. The high apparent diversity and extinction rate on the Adriatic Plate reflect oversampling of the Cassian Formation, a ‘liberation Lagerstätte’^57^, whereas the lower values in Siberia and South China likely result from the limited number of sampled fossil localities and the concentration of available occurrences in low-diversity shales. Other regional extinction and extirpation spikes, such as those in the Olenekian and Ladinian of Siberia and the Anisian of South China, also closely correspond to reductions in sampling (Fig. 4C, D).

These examples show how regional diversification patterns can be distorted by a combination of range dynamics, sampling bias, and tectono-sedimentary evolution, as also illustrated by a recent study on brachiopods^58^. By integrating occurrences across plates and environments, global analyses dampen region-specific biases that confound local records, thereby recovering a clearer and more temporally consistent macroevolutionary signal.

### Implications for estimating diversification dynamics across spatial scales

Both global and regional perspectives have distinct advantages and limitations. Diversification dynamics observed at different spatial and temporal scales may reflect different evolutionary processes^13^. Local and regional datasets can have relatively high temporal resolution, allowing their dynamics to capture fine-scale controls on diversity, as well as evidence for biotic interactions and local environmental conditions^15,59,60^. By contrast, global patterns are more likely to reflect large-scale mechanisms regulating diversity, such as climatic change and continental breakup^10,61–64^. Moreover, regional diversity fluctuations contain signals of range dynamics and can therefore provide insights into the formation of spatial diversity gradients and biotic invasions^12,21,49,50^. Global patterns, in contrast, are less affected by immigration and extirpation because these processes occur only within the global range of a taxon. The most appropriate spatial scale depends on the scientific question, and integrating global and regional perspectives can provide a more complete understanding of evolutionary drivers. However, given the current incompleteness of fossil data, regional patterns remain difficult to interpret, constraining the macroevolutionary information that can be inferred from them.

Currently, estimating regional diversification rates remains challenging for both biological and methodological reasons. First, fluctuations in regional diversity reflect the combined effects of diversification and range dynamics. These processes can have different temporal-spatial patterns^48–50^ (Fig. 4), and should be distinguished when assessing their drivers. The most widely used analytical methods, including phylogenetic tree-based and fossil occurrence-based approaches, were primarily developed for processes operating at global scales^23^ (but see refs. ^65,66^). Although diversification and range dynamics can sometimes be separated analytically^48–50^, the low sample sizes of fossil occurrences in many regional datasets reduce the statistical power and reliability of regional diversification rate estimates^22^.

Second, regional tectono-sedimentary history and fossil sampling complicate the detection of diversification drivers. Accounting for these processes is not straightforward because the stratigraphic distribution of fossils is non-random and strongly controlled by local environments and their sea-level-induced shifts^35,40^, and no single sampling proxy can fully capture these biases^34,67,68^. A range of analytical tools has been developed to mitigate temporal sampling bias in diversification rate estimation^4,69–74^, but these cannot yet fully accommodate the full suite of biases. These approaches can reduce the risk of inferring spurious rate shifts, or Type I errors, in which an apparent origination or extinction pulse is detected even though no such biological event occurred. For example, a decline in fossil sampling within an interval may produce an apparent extinction pulse, whereas sampling-corrected methods may weaken this signal or express the associated uncertainty through broad confidence intervals. However, these methods are less able to resolve Type II errors arising from false absences, in which genuine origination or extinction events are not detected because fossil records are sparse or discontinuous. For instance, the end-Triassic extinction is barely detected in South China because marine regression greatly reduced the availability of the marine fossil record beforehand (Fig. 4D). Consequently, the extent to which apparent regional diversification dynamics reflect intrinsic biological drivers remains uncertain.

Global patterns are also subject to temporal and spatial sampling bias^13,14,24,25,39,75–78^. Nevertheless, by integrating information from multiple incomplete regional records, global datasets generally record more true originations and extinctions and can partly mitigate the effects of low sample size and local sedimentary shifts encountered in regional studies, and as a result, shared large-scale signals may be retained while some asynchronous regional noise is dampened. However, the performance of global diversification rate estimates depends on the biogeographic structure of the clade under study. When interregional exchange is frequent and biogeographical differentiation is relatively weak (e.g., marine animals with high dispersal ability like foraminifers^50,79^, particularly in globally warm climates^64^), larger spatial coverage enhances fossil record completeness, improves estimates of true origination and extinction times, and increases the reliability of diversification-rate estimates. Conversely, in systems characterised by strong provincialism and regionally distinct diversification histories (e.g., terrestrial animals^75,76,80^), aggregating regional datasets can provide little improvement in sampling completeness. In such cases, global analyses may capture only the largest-scale events shared across regions (e.g., mass extinctions). In reality, no clade is completely uniform in biogeography, and the spatial coverage of fossil data may vary through time^13,17^. Different spatial-standardisation methods have been developed to reduce apparent rate variation caused by changes in spatial coverage, allowing fairer comparisons of diversity patterns among regions and time bins^14,17,19,75,76^. However, these methods cannot account for sedimentary and sampling biases that shape regional diversity fluctuations. Moreover, by substantially reducing sample sizes, spatial standardisation may increase the risk of Type II errors.

In summary, global and regional approaches are complementary, but their inferential power is asymmetrical under the constraints of the fossil record. At regional scales, reconstructing diversification dynamics and identifying their drivers remain limited by uneven sampling, sedimentary biases, and the overprinting effects of range dynamics. Analyses must navigate inherent trade-offs between spatial detail and temporal robustness, and between capturing local variability and recovering generalisable macroevolutionary patterns. For many clades, regional diversification signals remain too incomplete to support strong inferences. Integrative global analyses, however, can partially overcome these limitations by averaging across regions and environments, thereby isolating the shared macroevolutionary signal. Advancing beyond this asymmetry will require both increased fossil sampling and the development of analytical approaches that explicitly model and separate the interacting effects of diversification, dispersal, and sampling. Such advances are essential for moving from descriptive patterns toward a mechanistic understanding of biodiversity dynamics through deep time.

## Methods

### Fossil occurrence data

Post-Cambrian fossil occurrences of Bivalvia, Brachiopoda, and Cephalopoda were downloaded from the Paleobiology Database (PBDB) on 26/05/2026. The URLs used to download the datasets are provided in the Supplementary Material. Terrestrial records indicated by the ‘environment’ field in PBDB were discarded. Previous studies have shown that macroecological and macroevolutionary patterns may vary among taxonomic levels^81,82^. Given the greater completeness of genus-level data relative to species-level data^83,84^, and their widespread use in palaeontological studies, all analyses were carried out at the genus level. Subgeneric names were removed and only genus names were retained. Occurrences that cannot be assigned to a genus and those with ambiguous generic assignments (i.e., primary generic resolution noted by cf., aff., ?, informal, ex.gr., “”) were excluded. The PBDB is known to contain taxonomic errors that may affect estimates of diversification rates^85–87^. We filtered the PBDB data using the *pacmacro_ranges* function in fossilbrush R package^87^ (v1.0.6) to reduce the influence of taxonomic errors and wastebasket taxa on rate estimation. We applied the default settings and discarded occurrences falling outside the 90% density intervals of genus-level stratigraphic ranges. Although this procedure cannot eliminate all errors, random errors are unlikely to generate large-scale systematic biases^85^, particularly because our analyses compare regional and global patterns derived from the same underlying dataset.

We assigned fossil occurrences to stratigraphic stages using the divDyn R package^52^ (v0.8.3). Palaeo-coordinates of occurrences were reconstructed with the rgplates R package^88,89^ (v0.6.1) using the PALEOMAP rotation model^90^. We used the ‘geoplates2’ field in the PBDB data as an indicator of regions, which records the plate IDs used in the GPlates software^88^ under the PALEOMAP model^90^ (Fig. S1). Plate IDs in GPlates define groups of geological features that share the same reconstructed motion through time^88^. Although they do not correspond exactly to physical tectonic plates, they may represent the motion of features located on those physical plates. Plates were employed as spatial units because: (1) they allow regional fossil records to be consistently tracked through time, which is important when estimating regional diversification dynamics^16,17,22^; (2) fauna on different plates tend to share a lower compositional similarity and marine invertebrate biogeographic regions often coincide broadly with major plates^43,91^; and (3) the sizes of fossil-bearing areas on plates are comparable to those of regional units analysed in some previous studies^16,19^ (Fig. S1). Occurrences that could not be assigned to a stratigraphic stage or plate were discarded. After data cleaning, a total of 157,231, 130,418, and 89,936 occurrences in 2472, 3324, and 3679 genera were retained for the bivalve, brachiopod, and cephalopod datasets, respectively. All subsequent analyses were performed on these occurrences without accounting for the collections to which they belonged.

### Regional events

Regional events were defined as first and last appearances of genera on a given plate. These events were then classified in two downstream analyses.

In the first analysis, we quantified the extent to which regional events reflect range dynamics (immigrations and extirpations) rather than diversification dynamics (origination and extinction rates). To do so, we compared the timing of each regional event with the corresponding global first and last appearances of the same genus. Following other fossil-based studies^12,48,92^, global first and last appearances were used as approximations of taxonomic origination and extinction, although these dates are affected by preservation and sampling. If a genus first appeared on a plate later than its global origination, that regional appearance was interpreted as an immigration into the focal plate. Similarly, regional disappearances earlier than final global extinctions were treated as regional extirpations. It should be noted that we referred to these events as immigrations and extirpations for simplicity, but they could also be the result of preservation and sampling gaps. The numbers of immigrations and extirpations were calculated for each plate-stage combination. We then quantified their proportions relative to all regional events using two approaches: (1) the weighted ratio, defined as the total number of immigrations and extirpations across all plates and stages divided by the total number of regional events, and (2) the slope of a linear regression between the number of immigrations and extirpations and the number of regional events on each plate and stage. The intercept of the linear fit was forced to zero because neither immigrations nor extirpations can occur in the absence of regional events. To reduce noise associated with small sample sizes, only plate-stage combinations containing more than 100 occurrences were considered in the analyses. We first classified regional events using occurrences from all plates, and then retained only those plate-stage combinations that met this threshold when calculating the proportions of different event types. Consequently, 26,480 of 157,231 bivalve occurrences, 20,178 of 130,418 brachiopod occurrences, and 20,267 of 89,936 cephalopod occurrences were excluded from the calculation of these proportions (Fig. S2). However, the classification of regional events was performed without this filter. The first and last appearances of a genus are sometimes recorded on multiple plates within the same stage. In such cases, we cannot determine which occurrences represent the earliest or latest records, and these regional events were not counted as range shifts. This treatment will artificially bias estimates of range shifts downward.

In the second analysis, we assessed the impact of fossil sampling on the estimates of regional diversification rates by testing the correspondence between regional events and the changes in the availability of fossil localities. Changes in the spatial distribution of fossil localities were measured using grid-cell occupancy of fossil occurrences. Based on their palaeocoordinates, fossil occurrences were assigned to equal-area hexagonal cells with edge lengths of ~123 km, using the icosa R package^93^ (v0.12.0). A regional first appearance was classified as sampling-associated if the genus first appeared in cells that were not sampled in the preceding stage on the same plate. A regional last appearance was considered sampling-associated if all cells occupied by a genus in one stage were unsampled in the subsequent stage and the genus disappeared from the plate. An illustration of our approach is provided in Figure S3. Although plates drift through time, the palaeogeographical positions of fossil localities do not change substantially between adjacent geological stages (ca. 5 Myr in duration) and were thus assumed to be constant. The number of sampling-associated regional events was calculated for every plate and stage. Again, the proportions of sampling-associated events were quantified using the same weighted ratio and linear-fit approaches described above. The threshold of 100 occurrences was also applied, thereby excluding data points from poorly sampled plate-stage combinations with only a few cells (Fig. S2). We also investigated the relationship between the proportion of sampling-associated events and plate size. Plate size was quantified for each time interval as the maximum great-circle distance between occurrences on a given plate, using the divDyn package^52^. Because the spatial extent of sedimentary areas varies through time, the estimated maximum great-circle distance of each plate also varies through time.

To compare the prevalence of sampling-associated events at regional and global scales, we also conducted the same analysis on the global dataset using the same method. Because the identification of sampling-associated events depends on grid cell size, we also performed a sensitivity analysis using larger cells with edge lengths of ~247 km.

### Region- and genus-specific sampling patterns

To assess the quality of the fossil record, we calculated sampling rates and sampling completeness for individual plates and the global dataset. Sampling rate was measured as the number of fossil occurrences per million years. For each fossil dataset and stage, we calculated the sampling rate of every genus by dividing its number of occurrences by the duration of the stage. The median across genera was then used to represent the sampling rate of that dataset in that stage. As a proxy for sampling completeness, we used the three-timer completeness metric^4^, calculated using the divDyn package^52^. The metric is defined as $\frac{{t}_{3}}{{t}_{3}+{t}_{{part}}}$, where *t*_*3*_ is the number of taxa sampled in three consecutive bins and *t*_*part*_ is the number of taxa sampled in the bins immediately before and after, but not within, the focal bin. A low value indicates that many taxa expected to occur in the focal bin were not sampled there, suggesting poor sampling completeness. The two metrics were calculated for each plate-stage combination and for the global dataset in each stage, and the results were plotted in Fig. 3. We further calculated the median and its 95% bootstrap interval, based on 100 bootstrap replicates, for each metric to provide overall estimates for the regional and global datasets (Table S6).

We also calculated genus-specific sampling rate, duration, and geographical range for genera in each animal group. Because the exact ages of fossil occurrences are uncertain and long stages may lead to overestimation of taxon durations, we randomly sampled occurrence ages within stage boundaries and calculated the interval between first and last appearances. This procedure was repeated 100 times for each genus, and the median value was retained as the duration. Taxonomic sampling rate was then calculated as the total number of occurrences of the genus across its duration divided by that duration. Geographic range size was quantified as the maximum great-circle distance of all occurrences of the genus in all stages, and calculated using the divDyn package^52^. After calculating these metrics for each genus, we estimated group-level medians for bivalves, brachiopods, and cephalopods. Uncertainty in these medians was assessed using bootstrap resampling. For each group, genera were resampled with replacement 100 times, and the median value of each metric was recalculated for each bootstrap replicate. We report the median of these 100 bootstrap medians, together with the 2.5% and 97.5% quantiles as the 95% bootstrap interval.

### Contrast between regional and global rates

To assess differences between global and regional diversification rates, we analysed the occurrences of marine bivalves, a well-established model system for macroevolution^92,94,95^. We focused on extinction because global mass extinction events provide a reference framework for comparing regional and global signals.

For the global dataset, we calculated per-capita extinction rates^96^ using the divDyn package^52^. For regional datasets, we calculated three analogous rates: (1) regional disappearance rate, in which all regional last appearances were considered without distinguishing final extinction from extirpation; (2) regional extinction rate, in which only regional last appearances that coincided with the global final extinction of the genus were considered^22^; and (3) regional extirpation rate, in which only regional last appearances that preceded the global final extinction of the genus were considered. For example, the regional per-capita extirpation rate in a given stage was calculated as $-{\mathrm{ln}}(\frac{{N}_{{through}}}{{N}_{{extirpation}}+{N}_{{through}}})$, where *N*_*through*_ is the number of genera that have occurrences on the focal plate before and after the focal stage, and *N*_*extirpation*_ is the number of genera that occurred on the focal plate before the focal stage, had their last occurrence on that plate within the focal stage, but had a last occurrence on other plates after the focal stage. We highlighted the rate variation from the Late Permian to earliest Jurassic, which encompasses two of the five mass extinctions^3^ (end-Permian mass extinction and end-Triassic mass extinction), on three plates with the most abundant fossil occurrences during this interval: Siberian craton, South China plate, and Adriatic (Apulian) plate in southern Europe. For each of the three regions, we also calculated the number of fossil-bearing hexagonal cells (with edge lengths of ~123 km) in each stage.

### Statistics and reproducibility

All analyses were conducted in R^97^ (v4.5.3). After data cleaning, the datasets comprised 157,231 occurrences representing 2,472 bivalve genera, 130,418 occurrences representing 3,324 brachiopod genera, and 89,936 occurrences representing 3,679 cephalopod genera. For calculations of regional event proportions, only plate-stage combinations containing more than 100 occurrences were included to reduce noise associated with small sample sizes. One-sided Mann-Whitney U tests were used to determine whether global sampling rates and three-timer completeness were greater than their regional counterparts. These metrics were also summarised using medians, with 95% bootstrap intervals calculated from 100 bootstrap replicates. Spearman’s rank correlation tests were used to assess the relationships of the plate-level proportion of range shifts with the proportion of endemic taxa, global origination and extinction rates, and plate size. The same test was used to examine the relationship between the proportion of sampling-associated events and plate size. Boxplots were used to compare genus durations, geographical ranges, and taxonomic sampling rates among bivalves, brachiopods, and cephalopods. For genus-level duration estimates, occurrence ages were randomly sampled within stratigraphic stage boundaries 100 times, and the median estimate was retained. Uncertainty in group-level medians of genus duration, geographical range, and taxonomic sampling rate was assessed by resampling genera with replacement 100 times and reporting the median bootstrap estimate together with the 2.5% and 97.5% quantiles. All figures and tables can be reproduced using the data and code provided. In addition to the packages described above, packages used for data manipulation and visualisation included chronosphere^98^(v0.6.1), scales^99^ (v1.4.0), dplyr^100^ (v1.2.1), and stringr^101^ (v1.6.0).

### Reporting summary

Further information on research design is available in the Nature Portfolio Reporting Summary linked to this article.

## Supplementary information

Supplementary Figs. and Tables

Reporting Summary

## Data Availability

Fossil occurrence data were downloaded from the Paleobiology Database (paleobiodb.org). All datasets analysed in this study are available on Zenodo^102^.

## Code availability

The code used for data analysis and figure generation is available on Zenodo^102^.
