# Supplementary material for: Global patterns are key to understanding macroevolution
Source: Commun Biol. 2026 Oct 8;9:1313. doi: 10.1038/s42003-026-11118-7 (PMC13650148; doi:10.1038/s42003-026-11118-7)
Supplement: Supplementary file 1 [file 42003_2026_11118_MOESM1_ESM.pdf]

Supplementary Information

Contents:

1. URLs used to download the PBDB dataset
2. Supplementary Figures S1–S11
3. Supplementary Tables S1–S6

1. URLs used to download the PBDB dataset

Bivalvia:
https://paleobiodb.org/data1.2/occs/list.csv?base_name=Bivalvia&taxon_reso=genus&interval=Ordovician,Cenozoic&pgm=gplates,scotese,seton&show=attr,class,ident,coords,paleoloc,lith,env

Brachiopoda:
https://paleobiodb.org/data1.2/occs/list.csv?base_name=Brachiopoda&taxon_reso=genus&interval=Ordovician,Cenozoic&pgm=gplates,scotese,seton&show=attr,class,ident,coords,paleoloc,lith,env

Cephalopoda:
https://paleobiodb.org/data1.2/occs/list.csv?base_name=Cephalopoda&taxon_reso=genus&interval=Ordovician,Cenozoic&pgm=gplates,scotese,seton&show=attr,class,ident,coords,paleoloc,lith,env

2. Supplementary Figures

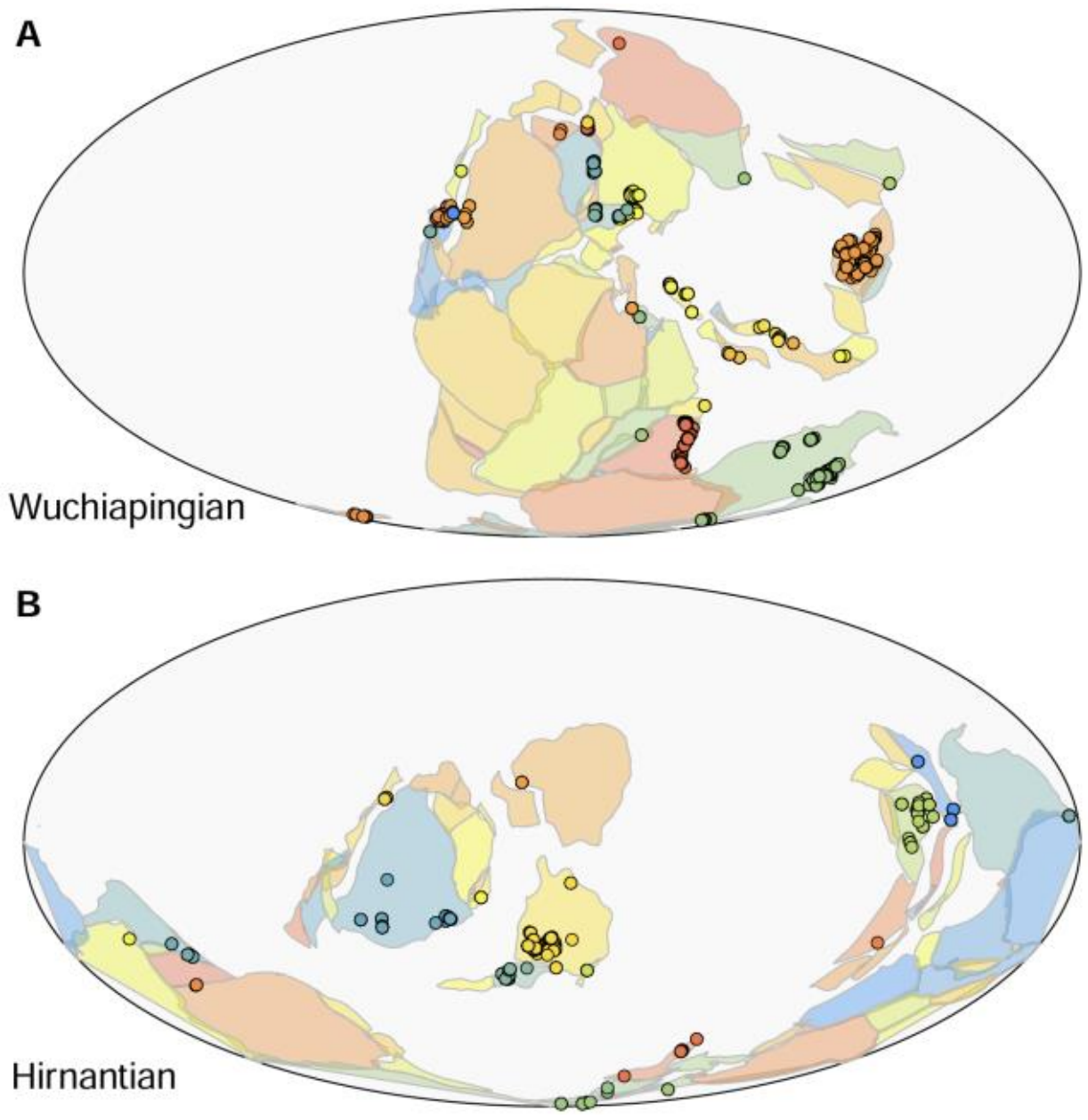

Figure S1. Palaeogeographical maps showing the spatial distribution of brachiopod fossil occurrences. A, Wuchiapingian; **B**, Hirnantian. Palaeogeographical reconstructions are based on the PALEOMAP model. Dots represent brachiopod fossil occurrences. Plates and their associated occurrences are distinguished by different colours.

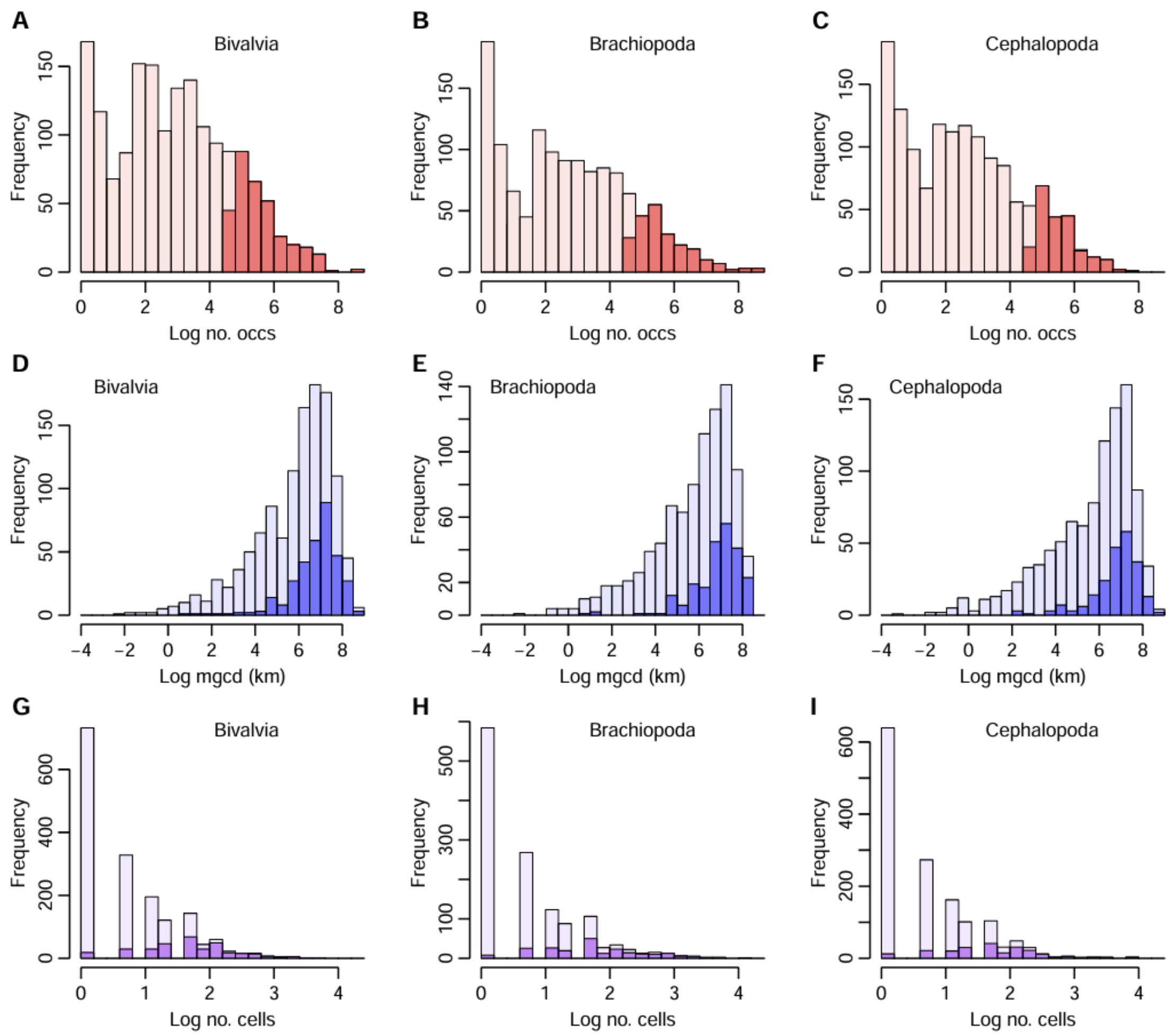

Figure S2. Distributions of the number of occurrences, the maximum great-circle distance of occurrences, and the number of sampled cells across plates. A–C show the log-transformed number of occurrences; **D–F** show the log-transformed maximum great-circle distance (mgcd; in km); **G–I** show the log-transformed number of sampled cells. The edge length of hexagonal grid cells is ~123 km. All metrics were calculated separately for each plate-stage combination; therefore, the same plate may have different values in different time intervals. Light colours represent all plate-stage combinations, whereas dark colours represent the subset remaining after plate-stage combinations with fewer than 100 occurrences were excluded. Many data points from small plates or plates with sparse fossil records were excluded from the calculation of the proportion of regional events.

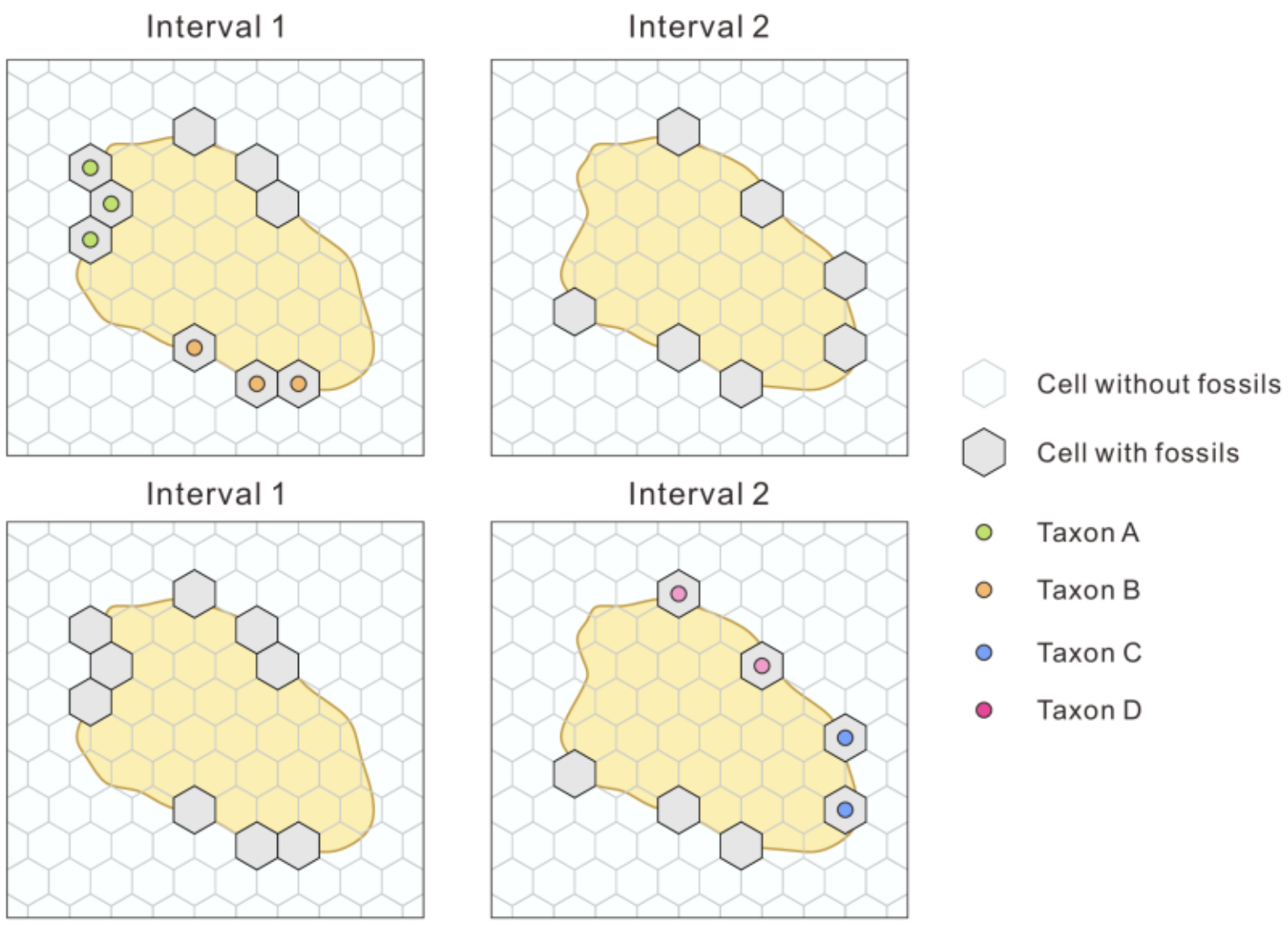

Figure S3. Schematic illustration of regional first and last appearances and changes in fossil-bearing cells between two adjacent time intervals on a plate. Taxa A and B have their last appearances on this plate in interval 1. For taxon A, all occupied cells in interval 1 are unsampled in interval 2, and its disappearance is classified as sampling-associated. For taxon B, some occupied cells in interval 1 are sampled in interval 2, and its disappearance is considered to be not sampling-associated. Taxa C and D have their first appearances in interval 2. For taxon C, all occupied cells in interval 2 are unsampled in interval 1, and its appearance is classified as sampling-associated. For taxon D, some occupied cells in interval 2 are sampled in interval 1, and its appearance is considered to be not sampling-associated.

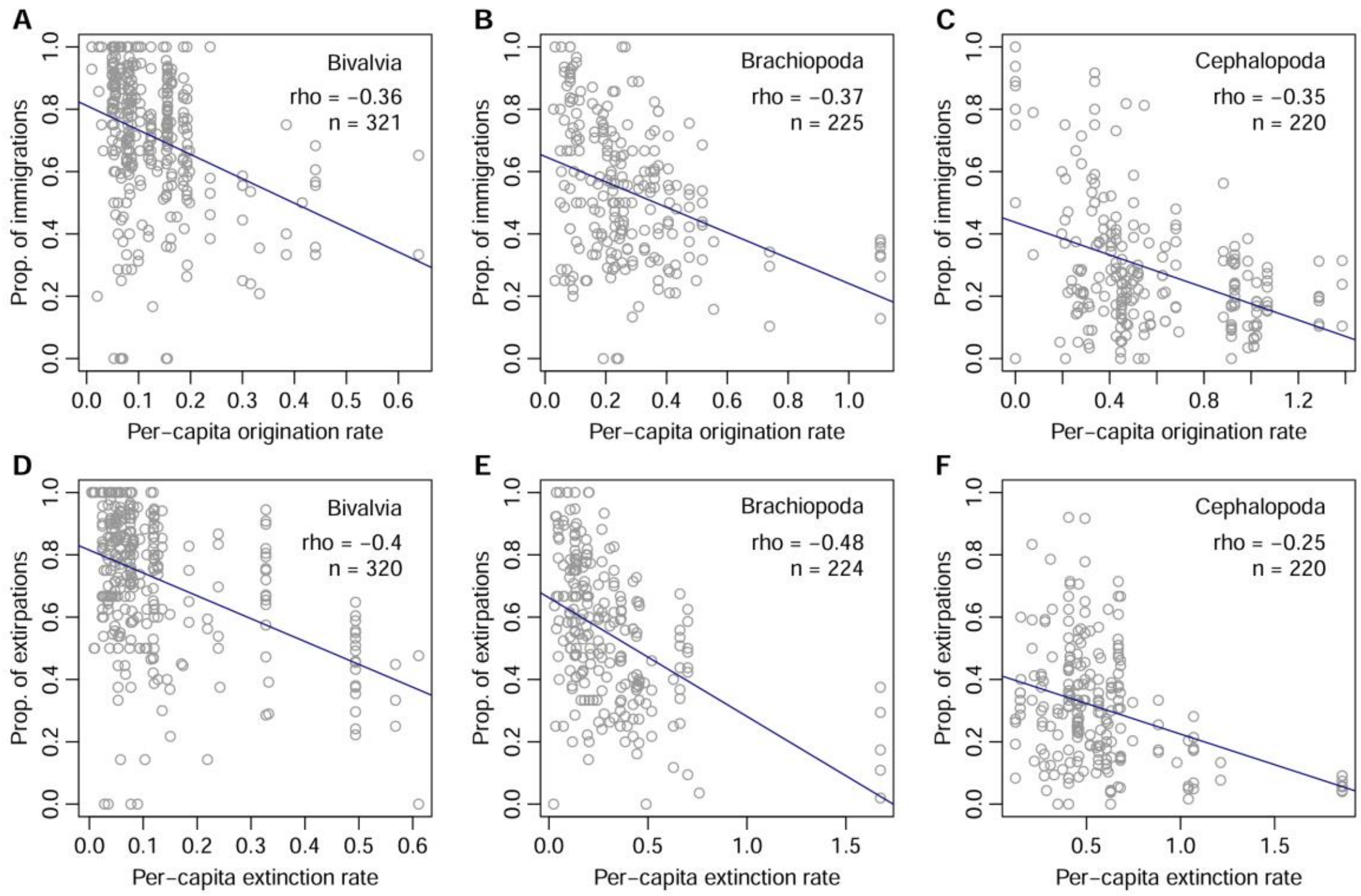

Figure S4. Plate-level proportions of immigration and extirpation decline with increasing global origination and extinction rates. A–C, relationships between the proportion of immigrations and the global per-capita origination rate in bivalves, brachiopods, and cephalopods, respectively. **D–F**, relationships between the proportion of extirpations and the global per-capita extinction rate in the same groups. For each plate and stage, the proportion of immigrations was calculated as the number of immigrations divided by the number of regional appearances; the proportion of extirpations was calculated analogously. Global rates are per-capita rates. Rho and n denote Spearman's rank correlation coefficient and sample size, respectively. Correlations are significant in all panels ($p < 0.0001$). Prop., proportion.

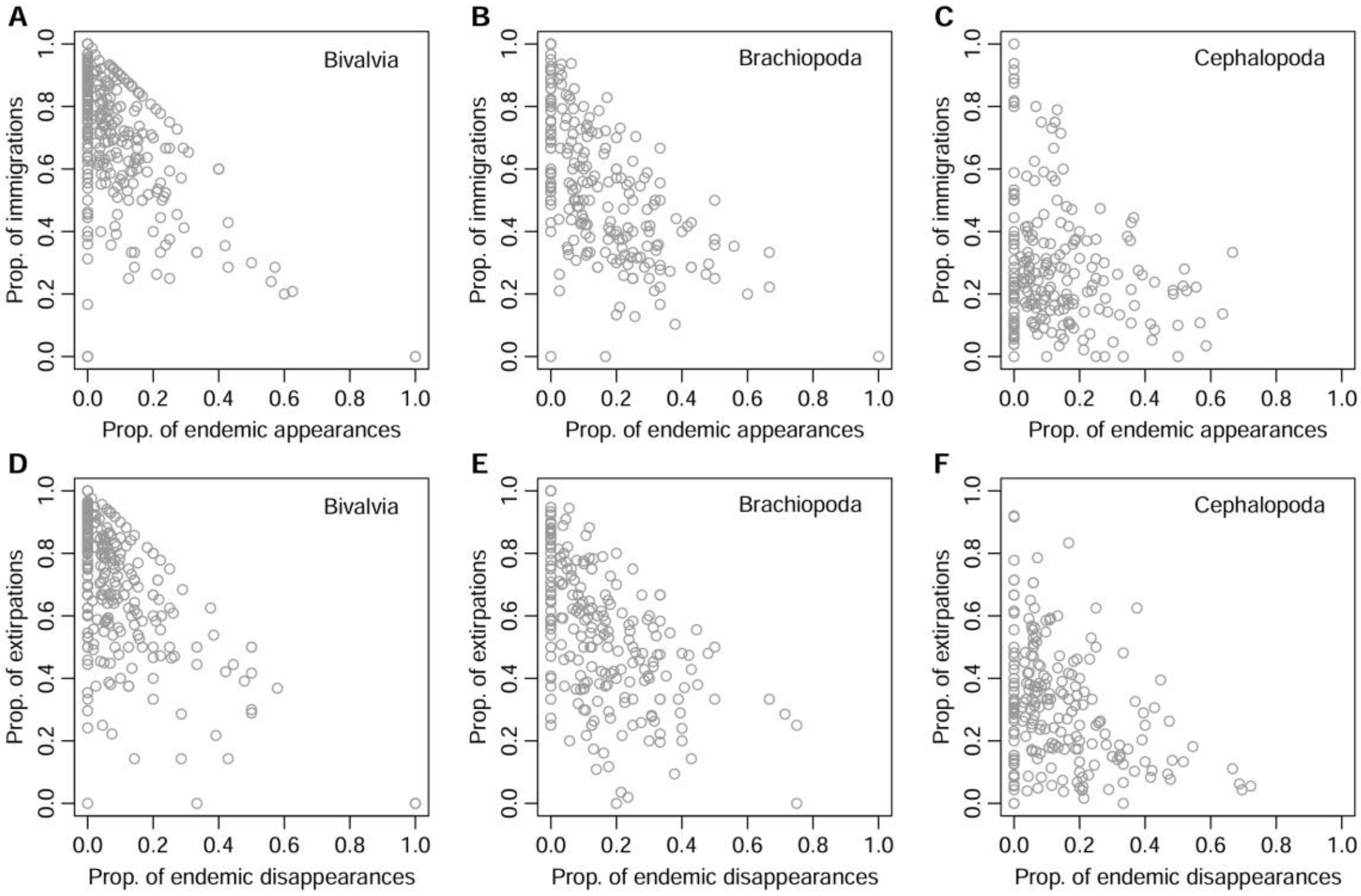

Figure S5. Plate-level proportions of immigration and extirpation decline as the proportion involving endemic genera increases. A–C, relationships between the proportion of immigrations and the proportion of appearances represented by endemic genera in bivalves, brachiopods, and cephalopods, respectively. **D–F**, relationships between the proportion of extirpations and the proportion of disappearances represented by endemic genera in the same groups. Endemic genera were defined here as those reported from only one plate. For each plate and stage, the proportion of endemic disappearances was calculated as the number of disappearances of endemic genera divided by the total number of disappearances; the proportion of endemic appearances was calculated analogously. The calculation of the proportion of regional immigrations and extirpations see the legend of Figure S4. Plates with higher proportions of endemic taxa tend to have lower proportions of detected range shifts because the identification or range shifts were based on comparison between regional and global datasets. Prop., proportions.

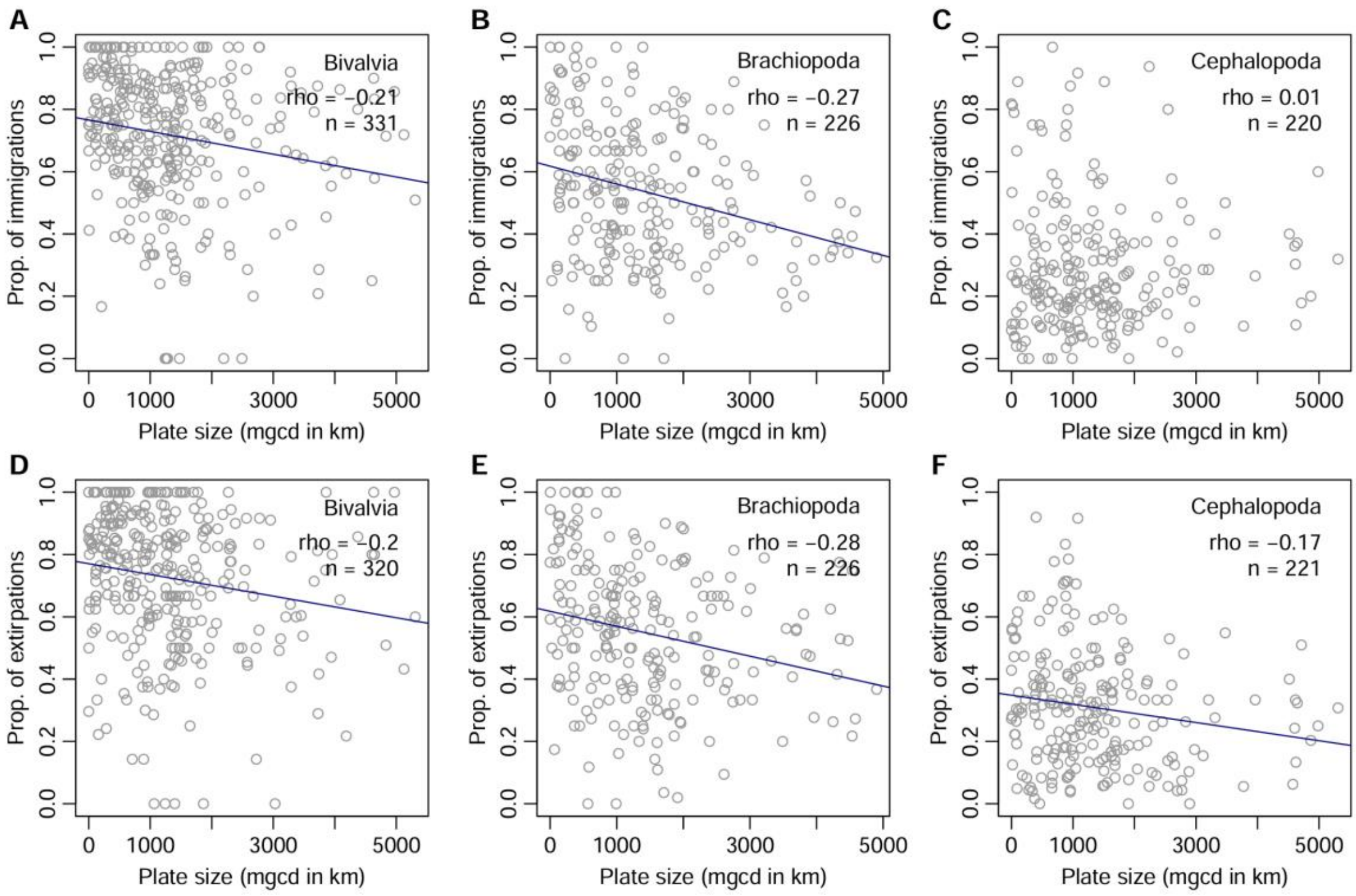

Figure S6. Plate-level proportions of immigration and extirpation decline with increasing plate size. A–C, relationships between the proportion of immigrations and plate size in bivalves, brachiopods, and cephalopods, respectively. **D–F**, relationships between the proportion of extirpations and plate size in the same groups. Plate size was quantified as the maximum great-circle distance between fossil occurrences recorded on a plate within each stage. The calculation of the proportion of regional immigrations and extirpations see the legend of Figure S4. Rho and n denote Spearman's rank correlation coefficient and sample size, respectively. Correlations are significant in all panels (Spearman's correlation, $p < 0.01$), except panel C ($p = 0.87$). Blue lines indicate linear regression fits for significant correlations. Prop., proportions.

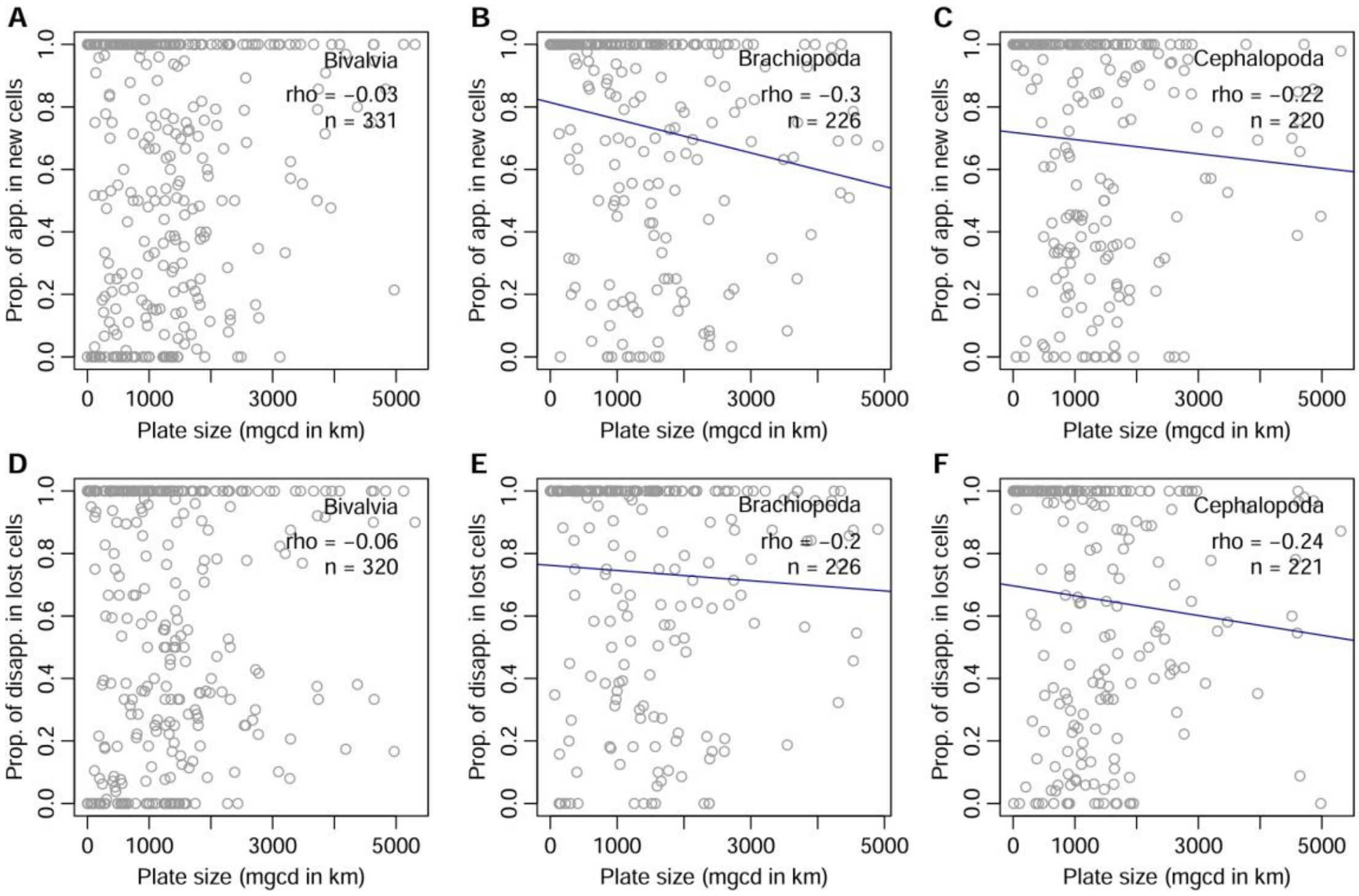

Figure S7. Relationship between the proportion of sampling-associated regional events and plate size. A–C, relationships between the proportion of sampling-associated appearances and plate size in bivalves, brachiopods, and cephalopods, respectively. **D–F**, relationships between the proportion of sampling-associated disappearances and plate size in the same groups. For each plate-stage combination, the proportion of sampling-associated events was calculated as the number of sampling-associated events divided by the total number of regional events. Plate size was quantified as the maximum great-circle distance between fossil occurrences recorded on a plate within each stage. Rho and n denote Spearman's rank correlation coefficient and sample size, respectively. Correlations are significant (Spearman's correlation, $p < 0.01$), except panels A and D ($p = 0.59$, 0.26, respectively). Blue lines indicate linear regression fits for significant correlations. Prop., proportions; app., appearances, disapp., disappearances.

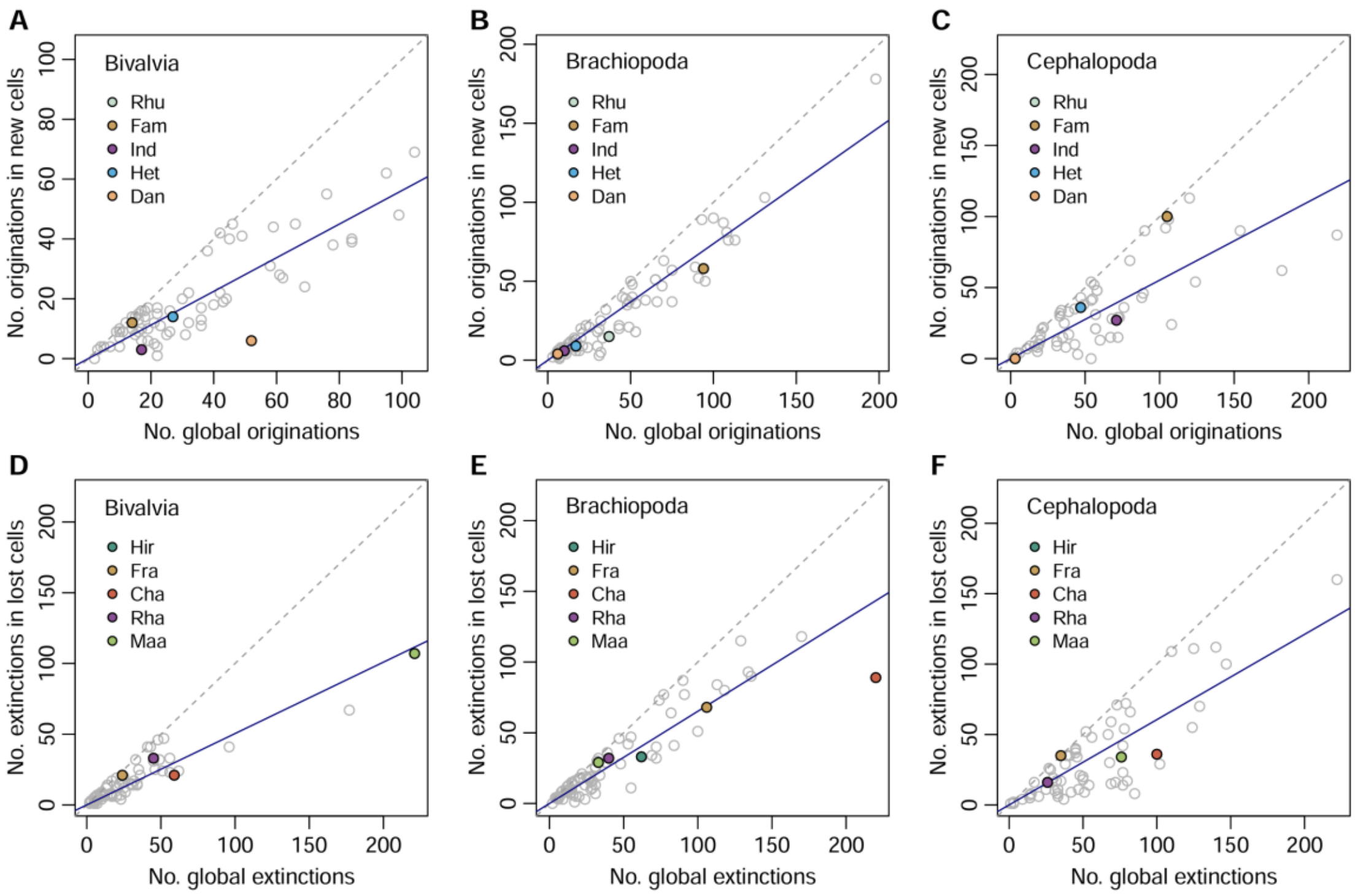

Figure S8. Relationship between the number of global originations/extinctions and the number of sampling-associated global events (small cells). The edge length of hexagonal grid cells is ~123 km. **A–C**, numbers of originations and those appeared in newly sampled cells. **D–F**, numbers of extinctions and those occurring in previously occupied, but subsequently unsampled cells. Data from post-mass-extinction intervals in A–C and from mass extinction intervals in D–F are highlighted with coloured circles; grey circles denote all other intervals. Blue lines show least-squares fits with the intercept constrained to zero. Gray dashed lines have a slope of one. Abbreviations as in Figure 2.

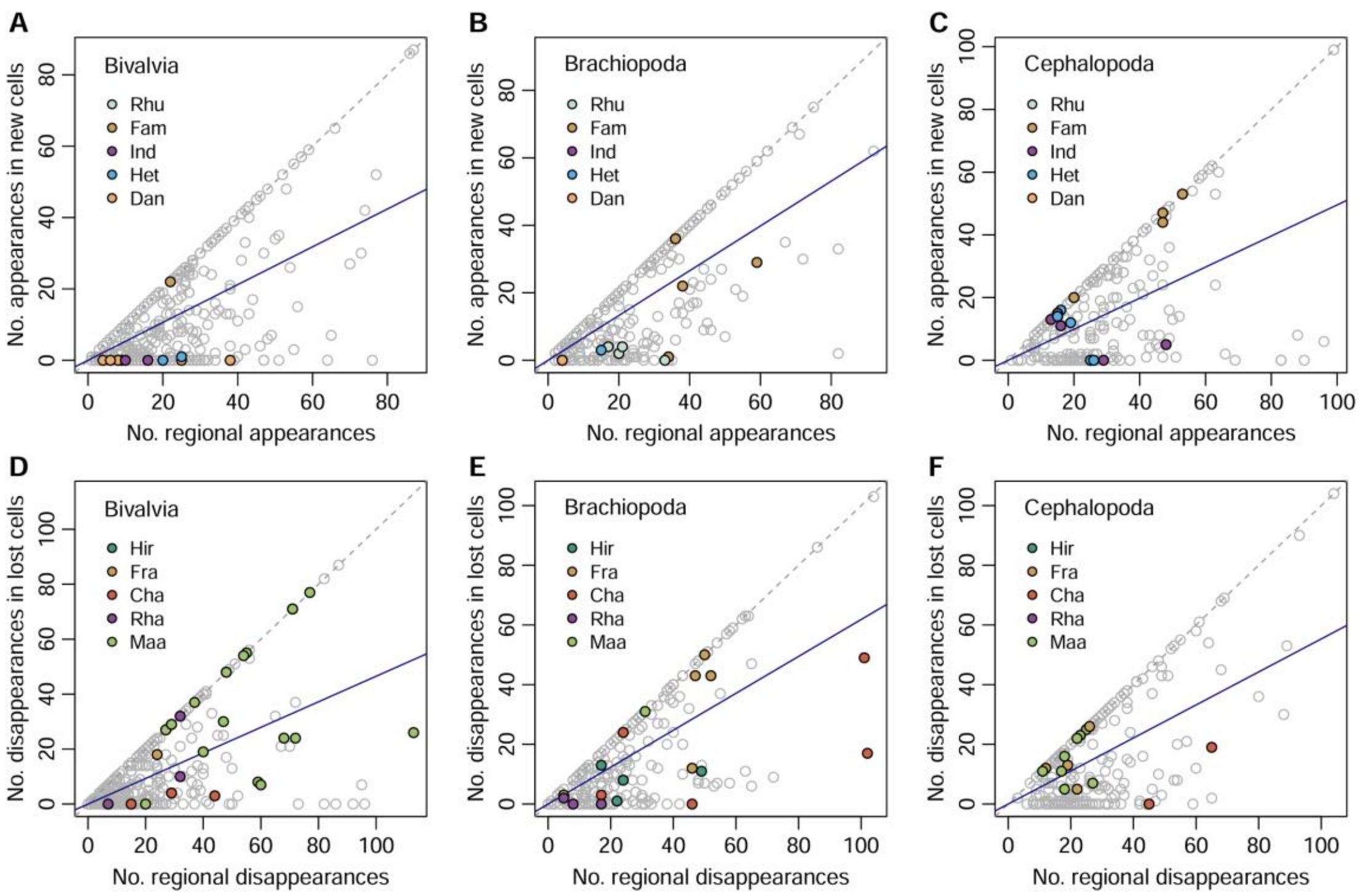

Figure S9. Relationship between the number of regional events and the number of sampling-associated events (large cells). This figure is analogous to Figure 2, but results are based on larger grid cells with edge length of ~247 km. **A–C**, numbers of regional first appearances and those appeared in newly sampled cells. **D–F**, numbers of regional last appearances and those occurring in previously sampled, but subsequently unsampled cells. Data from post-mass-extinction intervals in A–C and from mass extinction intervals in D–F are highlighted with coloured circles; grey circles denote all other intervals. Blue lines show least-squares fits with the intercept constrained to zero. Gray dashed lines have a slope of one. Abbreviations as in Figure 2.

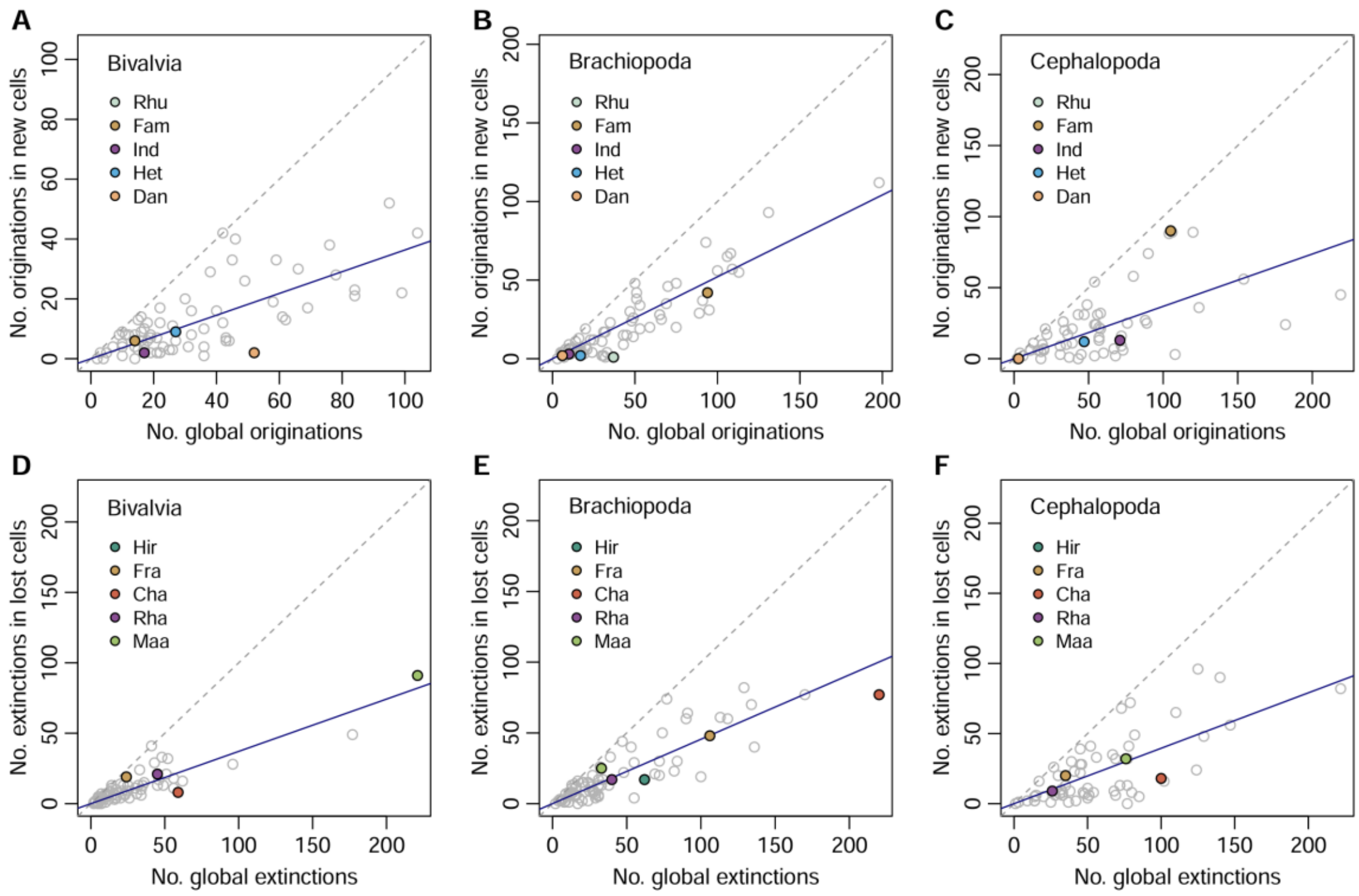

Figure S10. Relationship between the number of global originations/extinctions and the number of sampling-associated global events (large cells). This figure is analogous to Figure S5, but results are based on larger grid cells with edge length of ~247 km. **A–C**, numbers of originations and those appeared in newly sampled cells. **D–F**, numbers of extinctions and those occurring in previously occupied, but subsequently unsampled cells. Data from post-mass-extinction intervals in A–C and from mass extinction intervals in D–F are highlighted with coloured circles; grey circles denote all other intervals. Blue lines show least-squares fits with the intercept constrained to zero. Gray dashed lines have a slope of one. Abbreviations as in Figure 2.

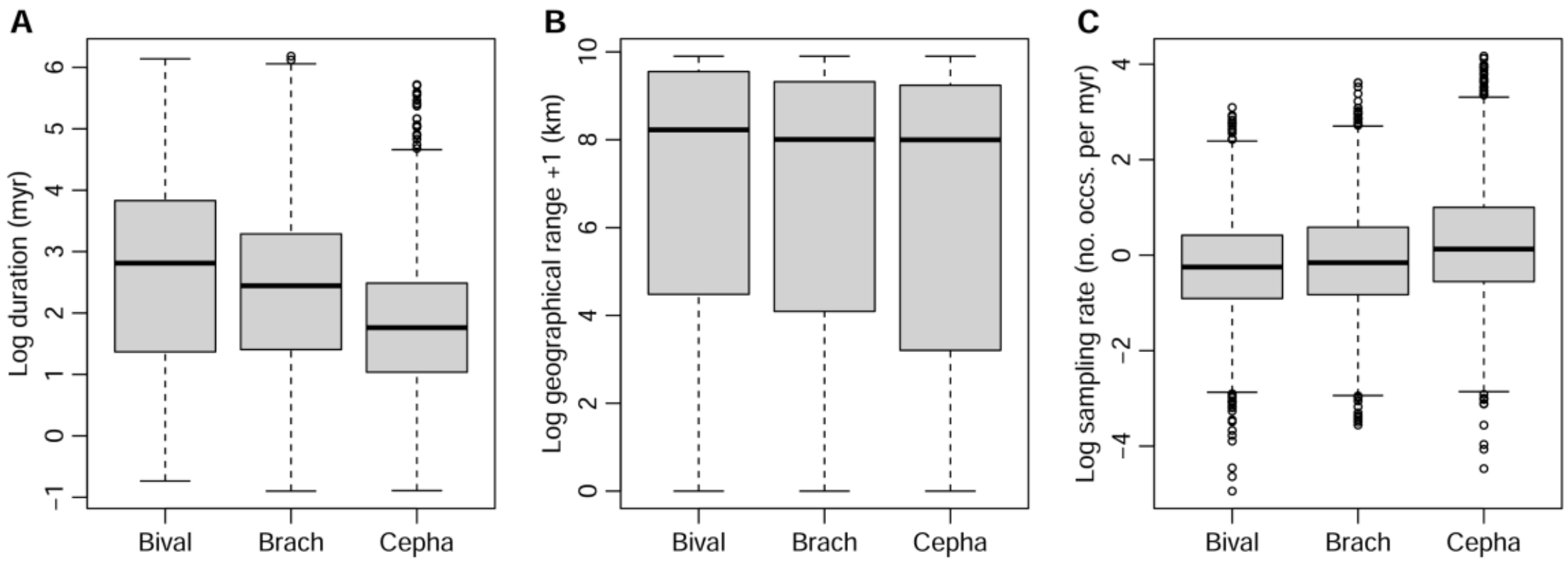

Figure S11. Durations, geographical ranges, and sampling rates of bivalve, brachiopod, and cephalopod genera. **A**, durations; **B**, geographical ranges; **C,** sampling rates. Geographical range is measured as the maximum great-circle distance among all occurrences. Sampling rate is number of fossil occurrences per million years. Duration and sampling rate are log-transformed, whereas geographical range is log(+1)-transformed. Median durations were 16.67 (95% bootstrap interval: 15.46–18.63) Myr for bivalves, 11.52 (11.00–11.97) Myr for brachiopods, and 5.82 (5.51–6.05) Myr for cephalopods. Median geographical ranges were 3,623 (95% bootstrap interval: 3,059–4,385) km for bivalves, 3,008 (2,597–3,416) km for brachiopods, and 2,981 (2,582–3,313) km for cephalopods. Median sampling rates were 0.78 (95% bootstrap interval: 0.75–0.81) for bivalves, 0.85 (0.82–0.89) for brachiopods, and 1.14 (1.09–1.19) for cephalopods. Sample sizes were n = 2472, 3324, and 3679 for bivalves, brachiopods, and cephalopods, respectively. Bival, bivalves; Brach, brachiopods; Cepha, cephalopods.

3. Supplementary Tables

Table S1. Proportions of regional immigrations and extirpations, measured by the weighted ratio and the slope of fitted lines. The weighted ratio was calculated as the total number of immigrations and extirpations across all plates and stages divided by the total number of regional events. For regional immigrations, 'other stages' refer to stages except for post-mass extinction stages; for regional extirpations, 'other stages' refer to stages except for mass extinction stages.

Dataset	Immigration		Extirpation	
	Weighted ratio	Slope	Weighted ratio	Slope
Bivalvia all stages	0.75	0.76	0.72	0.72
Bivalvia mass extinction stages	-	-	0.47	0.49
Bivalvia post-mass extinction stages	0.55	0.54	-	-
Bivalvia other stages	0.75	0.77	0.77	0.80
Brachiopoda all stages	0.55	0.53	0.52	0.47
Brachiopoda mass extinction stages	-	-	0.25	0.19
Brachiopoda post-mass extinction stages	0.43	0.44	-	-
Brachiopoda other stages	0.54	0.53	0.55	0.53
Cephalopoda all stages	0.26	0.23	0.28	0.25
Cephalopoda mass extinction stages	-	-	0.11	0.10
Cephalopoda post-mass extinction stages	0.08	0.06	-	-
Cephalopoda other stages	0.27	0.24	0.29	0.26

Table S2. Proportions of sampling-associated regional events, measured by the weighted ratio and the slope of fitted lines (small cells). The weighted ratio was calculated as the total number of immigrations and extirpations across all plates and stages divided by the total number of regional events. For regional appearances, 'other stages' refer to stages except for post-mass extinction stages; for regional disappearances, 'other stages' refer to stages except for mass extinction stages. The edge length of hexagonal grid cells is ~123 km.

Dataset	Appearances in new cells		Disappearances in lost cells	
	Weighted ratio	Slope	Weighted ratio	Slope
Bivalvia all stages	0.67	0.67	0.62	0.60
Bivalvia mass extinction stages	-	-	0.64	0.59
Bivalvia post-mass extinction stages	0.32	0.28	-	-
Bivalvia other stages	0.68	0.71	0.62	0.61
Brachiopoda all stages	0.78	0.80	0.78	0.79
Brachiopoda mass extinction stages	-	-	0.65	0.55
Brachiopoda post-mass extinction stages	0.64	0.63	-	-
Brachiopoda other stages	0.78	0.81	0.79	0.83
Cephalopoda all stages	0.71	0.71	0.68	0.72
Cephalopoda mass extinction stages	-	-	0.66	0.54
Cephalopoda post-mass extinction stages	0.81	0.83	-	-
Cephalopoda other stages	0.70	0.70	0.68	0.73

Table S3. Proportions of sampling-associated global events, measured by the weight ratio and the slope of fitted lines (small cells). The weighted ratio was calculated as the total number of immigrations and extirpations across all plates and stages divided by the total number of regional events. For originations, ‘other stages’ refer to stages except for post-mass extinction stages; for extinctions, ‘other stages’ refer to stages except for mass extinction stages. The edge length of hexagonal grid cells is ~123 km.

Dataset	Origination in new cells		Extinction in lost cells	
	Weighted ratio	Slope	Weighted ratio	Slope
Bivalvia all stages	0.57	0.56	0.58	0.51
Bivalvia mass extinction stages	-	-	0.52	0.49
Bivalvia post-mass extinction stages	0.32	0.23	-	-
Bivalvia other stages	0.59	0.57	0.60	0.51
Brachiopoda all stages	0.70	0.74	0.67	0.65
Brachiopoda mass extinction stages	-	-	0.54	0.47
Brachiopoda post-mass extinction stages	0.56	0.59	-	-
Brachiopoda other stages	0.70	0.75	0.69	0.71
Cephalopoda all stages	0.58	0.55	0.58	0.61
Cephalopoda mass extinction stages	-	-	0.51	0.44
Cephalopoda post-mass extinction stages	0.72	0.77	-	-
Cephalopoda other stages	0.57	0.54	0.58	0.62

Table S4. Proportions of sampling-associated regional events, measured by the weighted ratio and the slope of fitted lines (large cells). The weighted ratio was calculated as the total number of immigrations and extirpations across all plates and stages divided by the total number of regional events. For regional appearances, 'other stages' refer to stages except for post-mass extinction stages; for regional disappearances, 'other stages' refer to stages except for mass extinction stages. The edge length of hexagonal grid cells is ~247 km.

Dataset	Appearances in new cells		Disappearances in lost cells	
	Weighted ratio	Slope	Weighted ratio	Slope
Bivalvia all stages	0.49	0.53	0.47	0.47
Bivalvia mass extinction stages	-	-	0.61	0.59
Bivalvia post-mass extinction stages	0.13	0.12	-	-
Bivalvia other stages	0.50	0.54	0.45	0.42
Brachiopoda all stages	0.63	0.66	0.59	0.62
Brachiopoda mass extinction stages	-	-	0.47	0.43
Brachiopoda post-mass extinction stages	0.36	0.42	-	-
Brachiopoda other stages	0.64	0.68	0.61	0.66
Cephalopoda all stages	0.51	0.50	0.51	0.55
Cephalopoda mass extinction stages	-	-	0.56	0.43
Cephalopoda post-mass extinction stages	0.64	0.66	-	-
Cephalopoda other stages	0.50	0.49	0.50	0.56

Table S5. Proportions of sampling-associated global events, measured by the weighted ratio and the slope of fitted lines (large cells). The weighted ratio was calculated as the total number of immigrations and extirpations across all plates and stages divided by the total number of regional events. For originations, 'other stages' refer to stages except for post-mass extinction stages; for extinctions, 'other stages' refer to stages except for mass extinction stages. The edge length of hexagonal grid cells is ~247 km.

Dataset	Origination in new cells		Extinction in lost cells	
	Weighted ratio	Slope	Weighted ratio	Slope
Bivalvia all stages	0.37	0.36	0.41	0.37
Bivalvia mass extinction stages	-	-	0.40	0.40
Bivalvia post-mass extinction stages	0.17	0.12	-	-
Bivalvia other stages	0.38	0.37	0.41	0.35
Brachiopoda all stages	0.48	0.52	0.47	0.46
Brachiopoda mass extinction stages	-	-	0.40	0.37
Brachiopoda post-mass extinction stages	0.31	0.38	-	-
Brachiopoda other stages	0.49	0.53	0.48	0.48
Cephalopoda all stages	0.39	0.37	0.39	0.40
Cephalopoda mass extinction stages	-	-	0.33	0.29
Cephalopoda post-mass extinction stages	0.51	0.60	-	-
Cephalopoda other stages	0.39	0.35	0.39	0.40

Table S6. Sampling patterns in the fossil record. Values represent the median sampling rate or three-timer completeness for the regional or global datasets, with ranges indicating the 95% bootstrap intervals of the medians. Regional sampling rates are overestimated because intervals with zero fossil occurrences were not considered in the calculation.

	Sampling rate Median (95% bootstrap interval)		Three-timer completeness Median (95% bootstrap interval)	
	Global	Regional	Global	Regional
Bivalvia	0.71 (0.59–0.78)	0.26 (0.24–0.27)	0.86 (0.83–0.87)	0.66 (0.58–0.67)
Brachiopoda	0.71 (0.53–0.80)	0.27 (0.25–0.28)	0.85 (0.81–0.87)	0.69 (0.65–0.76)
Cephalopoda	0.65 (0.52–0.78)	0.27 (0.26–0.30)	0.81 (0.78–0.86)	0.71 (0.50–0.83)
