# Supplementary material for: Global patterns are key to understanding macroevolution
Source: Commun Biol. 2026 Oct 8;9:1313. doi: 10.1038/s42003-026-11118-7 (PMC13650148; doi:10.1038/s42003-026-11118-7)
Supplement: Supplementary file 2 [file 42003_2026_11118_MOESM2_ESM.pdf]

nature portfolio

Corresponding author(s): Zhen Guo

Last updated by author(s): Aug 5, 2026

Reporting Summary

Nature Portfolio wishes to improve the reproducibility of the work that we publish. This form provides structure for consistency and transparency in reporting. For further information on Nature Portfolio policies, see our Editorial Policies and the Editorial Policy Checklist.

Statistics

For all statistical analyses, confirm that the following items are present in the figure legend, table legend, main text, or Methods section.

n/a	Confirmed	
☐	☒	The exact sample size (n) for each experimental group/condition, given as a discrete number and unit of measurement
☐	☒	A statement on whether measurements were taken from distinct samples or whether the same sample was measured repeatedly
☐	☒	The statistical test(s) used AND whether they are one- or two-sided<br>*Only common tests should be described solely by name; describe more complex techniques in the Methods section.*
☐	☒	A description of all covariates tested
☐	☒	A description of any assumptions or corrections, such as tests of normality and adjustment for multiple comparisons
☐	☒	A full description of the statistical parameters including central tendency (e.g. means) or other basic estimates (e.g. regression coefficient) AND variation (e.g. standard deviation) or associated estimates of uncertainty (e.g. confidence intervals)
☐	☒	For null hypothesis testing, the test statistic (e.g. F, t, r) with confidence intervals, effect sizes, degrees of freedom and P value noted<br>*Give P values as exact values whenever suitable.*
☒	☐	For Bayesian analysis, information on the choice of priors and Markov chain Monte Carlo settings
☒	☐	For hierarchical and complex designs, identification of the appropriate level for tests and full reporting of outcomes
☐	☒	Estimates of effect sizes (e.g. Cohen's d, Pearson's r), indicating how they were calculated

Our web collection on statistics for biologists contains articles on many of the points above.

Software and code

Policy information about availability of computer code

Data collection	Fossil occurrence data were downloaded manually from the Paleobiology Database (PBDB). The URLs used for data retrieval are provided in the Supplementary Material.
Data analysis	All analyses were conducted in R v4.5.3. The following packages were used: fossilbrush v1.0.6, divDyn v0.8.3, icosa v0.12.0, rgplates v0.6.1, chronosphere v0.6.1, dplyr v1.2.1, scales v1.4.0, and stringr v1.6.0. All code is provided in the Supplementary Material and will be deposited in Zenodo.

For manuscripts utilizing custom algorithms or software that are central to the research but not yet described in published literature, software must be made available to editors and reviewers. We strongly encourage code deposition in a community repository (e.g. GitHub). See the Nature Portfolio guidelines for submitting code & software for further information.

Data

Policy information about availability of data

All manuscripts must include a data availability statement. This statement should provide the following information, where applicable:

- Accession codes, unique identifiers, or web links for publicly available datasets
- A description of any restrictions on data availability
- For clinical datasets or third party data, please ensure that the statement adheres to our policy

Fossil occurrence data were downloaded from the Paleobiology Database. All analysed data are provided in the Supplementary Material and will be deposited in Zenodo.

Research involving human participants, their data, or biological material

Policy information about studies with human participants or human data. See also policy information about sex, gender (identity/presentation), and sexual orientation and race, ethnicity and racism.

Reporting on sex and gender	NA
Reporting on race, ethnicity, or other socially relevant groupings	NA
Population characteristics	NA
Recruitment	NA
Ethics oversight	NA

Note that full information on the approval of the study protocol must also be provided in the manuscript.

Field-specific reporting

Please select the one below that is the best fit for your research. If you are not sure, read the appropriate sections before making your selection.

☐ Life sciences ☐ Behavioural & social sciences ☒ Ecological, evolutionary & environmental sciences

For a reference copy of the document with all sections, see nature.com/documents/nr-reporting-summary-flat.pdf

Ecological, evolutionary & environmental sciences study design

All studies must disclose on these points even when the disclosure is negative.

Study description	We evaluate the reliability of regional diversification rates (i.e., origination and extinction rates) using fossil occurrence data from three major groups of marine invertebrates (Bivalvia, Brachiopoda, Cephalopoda), which have relatively good fossil records and are widely used in macroevolutionary and palaeoecological studies. Using tectonic plates as regional units, we quantify the regional first and last appearances that do not represent true global originations and extinctions, and evaluate the relationship between regional diversity changes and shifts in the availability of fossil-bearing localities. We show that a substantial proportion of apparent plate-level generic turnover represents immigrations and extirpations rather than origination and extinction, and that regional diversity fluctuations are more strongly shaped by preservation, sampling, and tectono-sedimentary history than global patterns.
Research sample	The analysed data comprise Ordovician to Cenozoic fossil occurrences of Brachiopoda, Bivalvia, and Cephalopoda.
Sampling strategy	Only marine fossil records were included in this study. Unreliable occurrences were excluded (see Data exclusions).
Data collection	Fossil data were manually downloaded from the Paleobiology Database, and the URLs used for data retrieval are provided in the Supplementary Material.
Timing and spatial scale	The dataset spans the Ordovician to Cenozoic and is global in spatial scale. Occurrences were further assigned to geological plates to investigate regional diversification rates.
Data exclusions	Occurrences that cannot be assigned to a genus and those with ambiguous generic assignments (i.e., primary generic resolution noted by cf., aff., ?, informal, ex.gr., “”) were excluded. The ‘geoplate2’ field of the PBDB data was used as an indicator of palaeogeographical regions. Occurrences that could not be assigned to a stratigraphic stage or plate were discarded. We further filtered the data using the pacmacro_ranges function in fossilbrush R package to reduce the influence of taxonomic errors and wastebasket taxa on rate estimation. Default settings of the function were applied, and occurrences falling outside the 90% density intervals of genus-level stratigraphic ranges were discarded.

Reproducibility	All code and data used to generate the results are deposited in Zenodo (https://doi.org/10.5281/zenodo.21812688).
Randomization	We calculated genus-specific sampling rate and duration for genera. Because the exact ages of fossil occurrences are uncertain and long stages may lead to overestimation of taxon durations, we randomly sampled occurrences ages within stage boundaries and calculated the interval between first and last appearances. This procedure was repeated 100 times for each genus, and the median value was retained as the duration. Genus-specific sampling rate was then calculated as the total number of occurrences of the genus across its duration divided by that duration.
Blinding	Blinding was not applicable in this study. All analyses were conducted on publicly available fossil occurrence data using reproducible computational workflows.

Did the study involve field work? ☐ Yes ☒ No

Reporting for specific materials, systems and methods

We require information from authors about some types of materials, experimental systems and methods used in many studies. Here, indicate whether each material, system or method listed is relevant to your study. If you are not sure if a list item applies to your research, read the appropriate section before selecting a response.

Materials & experimental systems

n/a	Involved in the study
☒	☐ Antibodies
☒	☐ Eukaryotic cell lines
☐	☒ Palaeontology and archaeology
☒	☐ Animals and other organisms
☒	☐ Clinical data
☒	☐ Dual use research of concern
☒	☐ Plants

Methods

n/a	Involved in the study
☒	☐ ChIP-seq
☒	☐ Flow cytometry
☒	☐ MRI-based neuroimaging

Palaeontology and Archaeology

Specimen provenance	This study does not involve specimen-based research.
Specimen deposition	This study does not involve specimen-based research.
Dating methods	This study does not involve specimen-based research.

☐ Tick this box to confirm that the raw and calibrated dates are available in the paper or in Supplementary Information.

Ethics oversight	This study does not involve specimen-based research.

Note that full information on the approval of the study protocol must also be provided in the manuscript.

Plants

Seed stocks	NA
Novel plant genotypes	NA
Authentication	NA
